# Clinical Safety and Efficacy of Hyaluronic Acid–Niacinamide–Tranexamic Acid Injectable Hydrogel for Multifactorial Facial Skin Quality Enhancement with Dark Skin Lightening

**DOI:** 10.3390/gels11070495

**Published:** 2025-06-26

**Authors:** Sarah Hsin, Kelly Lourenço, Alexandre Porcello, Michèle Chemali, Cíntia Marques, Wassim Raffoul, Marco Cerrano, Lee Ann Applegate, Alexis E. Laurent

**Affiliations:** 1 Development Department, LOUNA REGENERATIVE SA, CH-1207 Geneva, Switzerland; s.hsin@louna-aesthetics.com (S.H.); k.lourenco@louna-aesthetics.com (K.L.); a.porcello@louna-aesthetics.com (A.P.);; 2 Plastic and Aesthetic Surgery Service, Centre Médical Lausanne Ouest, CH-1008 Prilly, Switzerland; m.chemali@cmlo.ch; 3 Plastic and Reconstructive Surgery, Ensemble Hospitalier de la Côte, CH-1110 Morges, Switzerland; wassim.raffoul@ehc.vd.ch; 4 Aesthetic Surgery Department, Clinique Entourage, CH-1003 Lausanne, Switzerland; m.cerrano@entourage.ch; 5 Regenerative Therapy Unit, Lausanne University Hospital, University of Lausanne, CH-1066 Epalinges, Switzerland; lee.laurent-applegate@chuv.ch; 6 Center for Applied Biotechnology and Molecular Medicine, University of Zurich, CH-8057 Zurich, Switzerland; 7 Oxford OSCAR Suzhou Center, Oxford University, Suzhou 215123, China; 8 Manufacturing Department, LAM Biotechnologies SA, CH-1066 Epalinges, Switzerland; 9 Manufacturing Department, TEC-PHARMA SA, CH-1038 Bercher, Switzerland

**Keywords:** aesthetic medicine, clinical trial, dermal fillers, facial aging, hyaluronic acid, hyperpigmentation, injectable therapeutics, niacinamide, skin texture, tranexamic acid

## Abstract

Facial aging is a complex process manifesting as skin hyperpigmentation, textural irregularities, and a diminished elasticity, hydration, and evenness of tone. The escalating demand for minimally invasive aesthetic interventions has driven the development of advanced hydrogel-based injectable formulations. This clinical study assessed the safety and efficacy of Hydragel A1, an injectable hydrogel containing hyaluronic acid (HA), niacinamide, and tranexamic acid (TXA), designed to simultaneously address multiple facets of facial skin aging. A cohort of 49 female participants underwent a series of objective and subjective assessments, including the Global Aesthetic Improvement Scale (GAIS), instrumental measurements (Antera 3D, Chromameter, Cutometer, Dermascan, Corneometer), and standardized photographic documentation at baseline (Day 0) and 14, 28, and 70 days post-treatment. The results demonstrated statistically significant improvements in skin hydration, texture, elasticity, and pigmentation following Hydragel A1 administration. Notably, no serious adverse events or significant injection site reactions were observed, confirming the favorable safety profile of the investigated device. Collectively, these findings underscore the potential of a combined HA, niacinamide, and TXA injectable formulation to provide a comprehensive approach to facial skin rejuvenation, effectively targeting multiple aging-related mechanisms.

## 1. Introduction

Facial aging is a dynamic and multifactorial process driven by the interplay of genetic, biological, and environmental factors, culminating in discernible alterations in skin appearance. Prominent indicators of this process include hyperpigmentation and textural irregularities, both intrinsically linked to the underlying biological mechanisms of aging. Specifically, hyperpigmentation arises from a dysregulation of melanin production, whereas textural degradation manifests as a reduction in skin elasticity, hydration, and surface homogeneity [1]. These changes collectively impact the perceived aesthetics of the skin, influencing not only physical appearance but also psychological well-being and social interactions. Consequently, there is an escalating demand for innovative and effective therapeutic interventions that comprehensively address these concerns.

Despite advancements in dermatological science, the therapeutic management of skin pigmentation and compromised skin texture remains challenging. Current standard treatments for skin aging predominantly rely on topical agents. For hyperpigmentation, hydroquinone remains a widely utilized treatment, alongside other agents such as tranexamic acid (TXA) and niacinamide [2,3]. The skin tone-correcting effects of TXA and niacinamide have been extensively studied and comparatively analyzed [3,4]. Concurrently, hyaluronic acid (HA) is considered a cornerstone in addressing skin texture, leveraging its hydrating and volumizing properties to restore cutaneous moisture balance and structural integrity [5]. While effective within their respective domains, these topical treatments primarily target superficial skin layers, often limited by suboptimal penetration and transient efficacy [6].

Recent dermatological research has explored injectable therapies as a potential avenue to overcome these limitations. Novel injectable formulations, encompassing advanced skin tone agents and refined HA-based products, offer the potential for enhanced dermal penetration and sustained improvements in skin appearance. For instance, injectable HA has demonstrated promising outcomes in restoring skin structure and hydration [7,8]. Simultaneously, there is growing interest in injectable containing skin pigmentation-regulating agents, which may provide a more precise modulation of melanin production and distribution [7,9]. However, the long-term efficacy, mechanisms of action, and potential for synergistic combination therapies of these injectables remain areas of active investigation.

Despite the increasing interest in injectable modalities, a gap persists in comprehensive solutions that simultaneously address both skin pigmentation and texture. The current landscape of injectable treatments often focuses on singular concerns, such as volumization, hydration, or skin tone correction. However, the interconnected nature of skin aging processes necessitates a multifaceted approach, targeting both skin tone and textural degradation in tandem, to achieve more holistic and enduring improvements. This is particularly salient given the growing recognition that visible signs of aging are not isolated phenomena but rather the result of complex interactions between diverse skin properties [1].

Hydragel A1, an injectable medical device product under clinical investigation, contains HA, TXA, and niacinamide, aiming to enhance overall skin quality and correct mild lines. The scientific rationale of this study is based on the postulate that a deeper, localized delivery of these agents may yield more profound and sustained enhancements in skin attributes and aesthetics. In addition to evaluating the clinical efficacy of Hydragel A1, this study also assesses the safety profile of the formulation, alongside patient satisfaction and quality-of-life improvements. Given the increasing demand for non-surgical, minimally invasive treatments that offer both aesthetic and psychological benefits, this research has the potential to significantly advance the field. The novelty of this study lies in the acquisition of human clinical data on an injectable device formulation containing HA, TXA, and niacinamide.

Specifically, this clinical trial evaluates the safety and effectiveness of Hydragel A1 in enhancing overall facial skin aesthetics. The anticipated findings are expected to provide valuable insights into the efficacy, safety, and practical applications of Hydragel A1, contributing to the advancement of skin aesthetics and the management of visible signs of aging. The primary aim of this clinical investigation was to rigorously determine the benefit/risk profile in the intended target populations and medical indications and to demonstrate the acceptability of that profile. Specific objectives to the study included evaluating the efficacy of the investigational medical device at 4 weeks (i.e., 28 days (D28)) and 10 weeks (i.e., 70 days (D70)) post-treatment and assessing its ease of administration, safety, and tolerance throughout the study period. 

## 2. Results and Discussion

### 2.1. Global Aesthetic Improvement Scales

**Figure 1:**
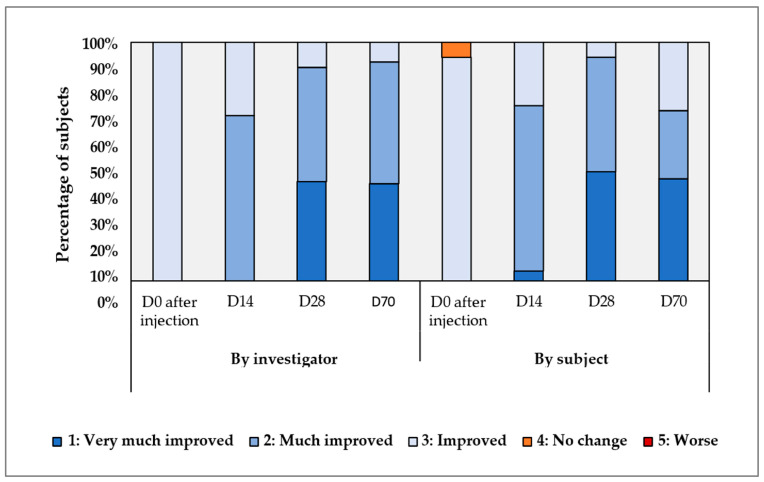
Results of IGAIS and SGAIS scores collected during the clinical study of the Hydragel A1 medical device. Detailed descriptions of the significance of the various grades are presented in Table S1. IGAIS, Investigator Global Aesthetic Improvement Scale; SGAIS, Subject Global Aesthetic Improvement Scale.

The results of the reported clinical study demonstrated substantial improvements in both the Investigator and Subject Global Aesthetic Improvement Scales (IGAIS and SGAIS) at various timepoints (i.e., Day 0, Day 14, Day 28, and Day 70) following the intradermal injections of Hydragel A1 and in comparison with Day 3 (D-3), which corresponds to the baseline skin condition prior to injection. Specifically, the improvements were observed consistently across both scales, as shown in Figure 1.

A detailed analysis revealed immediate improvements following the initial injection at Day 0. Specifically, 93% of participants reported “Improved” ratings according to the Subject Global Aesthetic Improvement Scale (SGAIS), while 100% were rated “Improved” by the injector, as documented by the Investigator Global Aesthetic Improvement Scale (IGAIS) (Figure 1). By Day 14, subsequent to the second product injection, a significant increase in the proportion of participants reporting “Much Improved” was observed. Specifically, 69.4% of participants self-assessed as “Much Improved”, a finding corroborated by the IGAIS, which yielded the same rating (Figure 1). At Day 28, during the initial post-injection follow-up visit, the improvements became more pronounced, with over 40% of participants (41.7% in IGAIS, 45.8% in SGAIS) categorized as “Much Improved” (Figure 1).

Only three participants reported “No Change” scores, all recorded at Day 0 in the SGAIS. Critically, no “Worse” responses were reported throughout the study in either the IGAIS or SGAIS (Figure 1). Across all timepoints, the IGAIS and SGAIS results demonstrated high consistency, with similar proportions of participants reporting improvement on both scales. The differences between timepoints were statistically significant (i.e., *p*-value < 0.001), with notable improvements observed from Day 0 and sustained effects through Day 70 (Table S2).

Overall, investigator assessments indicated that 100% of participants exhibited improvement as early as after the first product injection (Day 0), with these improvements sustained through Day 70. Regarding self-assessments, while three participants did not report improvement immediately after the first product injection, 100% of participants reported improvement from Day 14 onward. Four weeks post initial injection, 100% of participants (48/48) treated with Hydragel A1 were rated as improved by the investigator according to the GAIS. This proportion was significantly different from 40% (i.e., *p*-value < 0.001, Table S2). Furthermore, when investigator and subject evaluations were combined, the lower confidence interval for the proportion of participants showing improvement ranged from 85% to 94%. Overall, the proportion of recorded clinical success was significantly greater than 0.4 at all timepoints.

### 2.2. Clinical Photography Results

**Figure 2:**
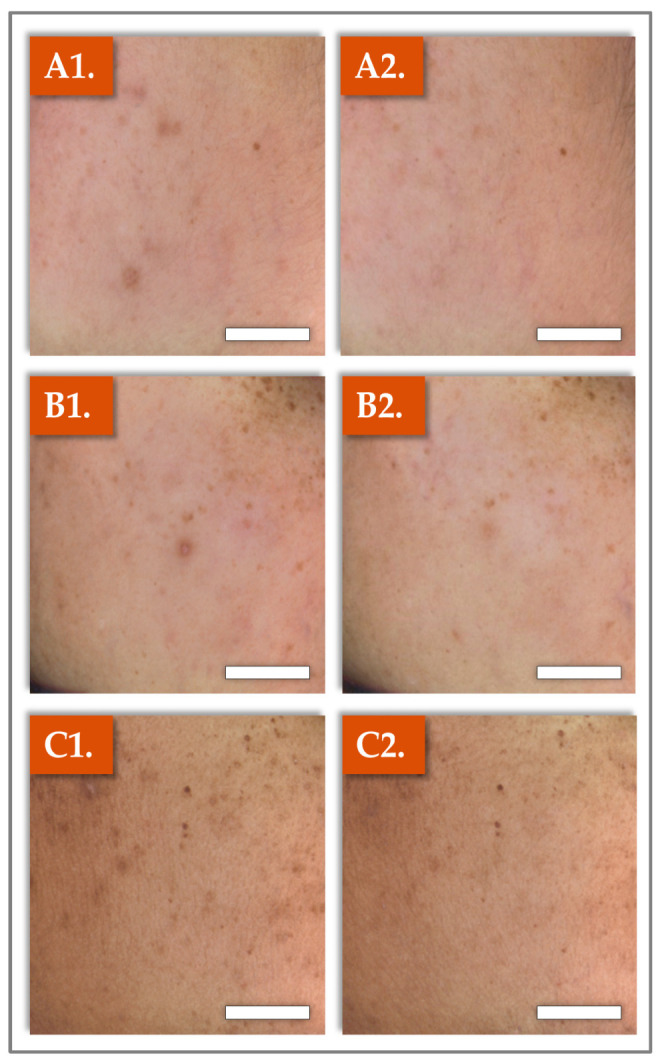
Images from three selected study patients, showing a visible improvement of skin radiance (i.e., from left [D–3], pre-baseline to right [D70]), both in terms of spot intensity and size of the dark spots. (**A1**,**A2**) Patient N° 17. (**B1**,**B2**) Patient N° 19. (**C1**,**C2**) Patient N° 26. Scale bars = 10 mm.

For macroscopic recording during patient monitoring, standardized photographs of selected areas of the cheeks were taken at pre-baseline (D–3; i.e., left column) and at the end of the study at D70 (i.e., right column; Figure 2).

The presented findings are representative of the average improvements observed in the study participants as a result of the administration of Hydragel A1 (Figure 2).

### 2.3. Antera 3D Results for Skin Texture and Roughness

**Figure 3:**
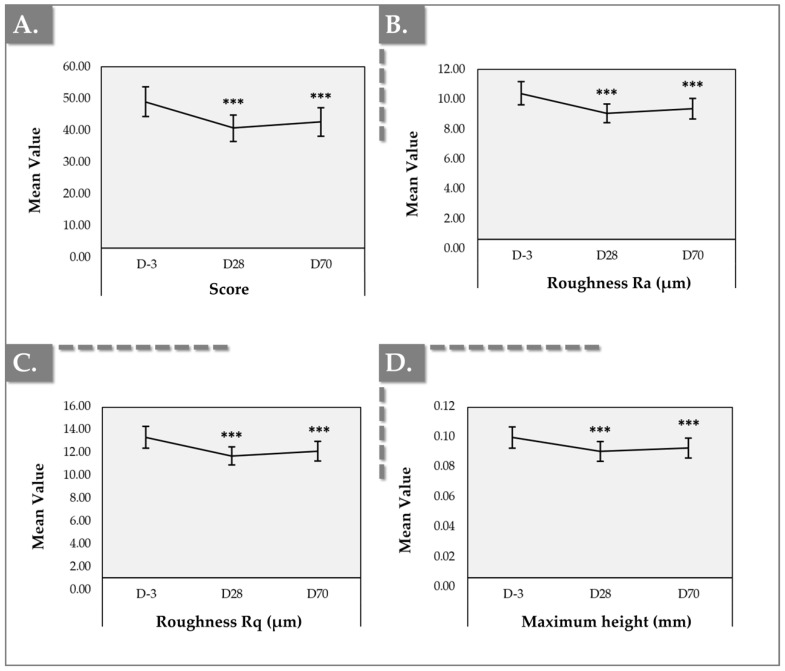
Clinical monitoring results using the Antera 3D device. (**A**) Texture score evolution. (**B**) Roughness “Ra” parameter evolution. (**C**) Roughness “Rq” parameter evolution. (**D**) Maximum height evolution. Error bars = 95% confidence interval. Statistical significance (i.e., *p*-value ≤ 0.001) was evidenced by three asterisks (***). Detailed statistical analysis results are reported in Table S3.

Instrumental measurements using Antera 3D demonstrated significant improvements in skin texture and roughness parameters following product administration, with changes sustained throughout both D28 and D70 timepoints. All parameters, including texture score, surface roughness (i.e., Ra and Rq) and maximum height, showed statistically significant improvements compared to baseline values at D-3 (Figure 3 and Figure S1, Table S3).

Firstly, the mean texture score decreased from a mean value of 48.50 ± 16.97 at baseline to 39.78 ± 14.92 at D28, a result sustained at D70 (i.e., mean value of 41.84 ± 16.27), indicating an improvement in skin texture (i.e., *p*-value < 0.001, Wilcoxon signed-rank test; Table S3). The reduction in texture score corresponded to a 13.47% improvement at D70 compared to baseline (Table S3) and 100% of the subjects showed a reduction at D70, thus showing an improvement.

Secondly, surface roughness (Ra) showed a significant reduction from a mean of 10.32 ± 2.85 µm at baseline to 8.90 ± 2.27 µm at D28 and 9.23 ± 2.27 µm at D70, showing a statistically significant decrease in texture inhomogeneity (i.e., *p*-value < 0.001, Wilcoxon signed-rank test; Table S3). This reduction represented a 13.77% improvement at D28, and 10.59% improvement at D70 compared to baseline (Table S3). Additionally, 98% of the subjects showed a reduction at D28 and 100% at D70, thus showing an improvement. These results were correlated by the root mean square roughness (Rq), which demonstrated a statistically significant improvement with a mean decrease from 13.22 ± 3.60 µm at baseline to 11.45 ± 2.95 µm at D28, a result sustained at D70 with a mean value of 11.90 ± 3.25 µm (Table S3). The improvement in texture confirmed the Ra results, with a calculated decrease in texture of 13.37% at D28 (i.e., *p*-value < 0.001, Wilcoxon signed-rank test) and of 9.98% at D70 (i.e., *p*-value < 0.001, Paired *t*-test; Table S3).

Thirdly, the maximum height of skin surface features showed a mean decrease from 0.10 ± 0.03 mm at D-3 to 0.09 ± 0.02 mm at D28 and D70, with minimum and maximum values ranging from 0.06 mm to 0.17 mm at D-3 and from 0.04 mm to 0.15 mm at D70. These results represented an improvement of 7.48% at D70 compared to baseline (i.e., *p*-value < 0.001, Paired *t*-test; Table S3).

**Figure 4:**
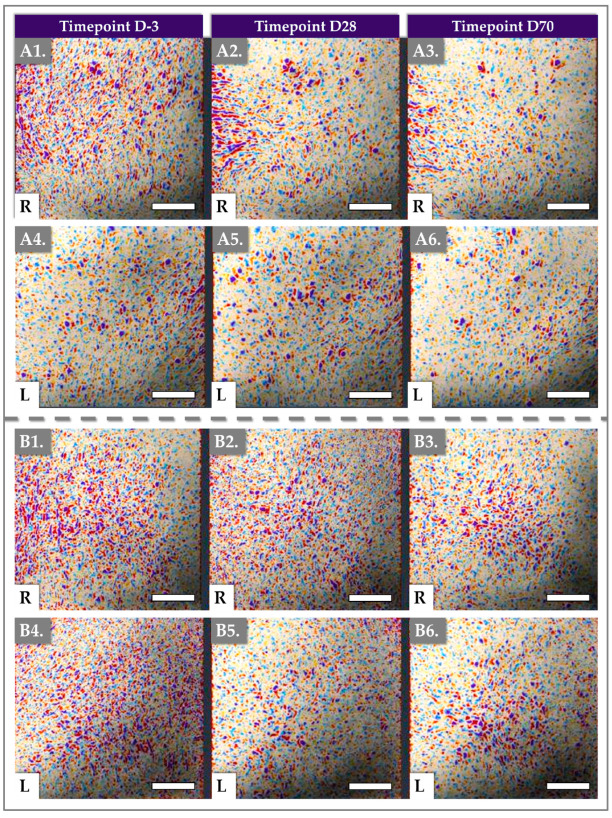
Results of Antera 3D imaging at various timepoints of the clinical study. (**A1**–**A3**) Imaging of the right cheek for patient N° 4. (**A4**–**A6**) Imaging of the left cheek for patient N° 4. (**B1**–**B3**) Imaging of the right cheek for patient N° 37. (**B4**–**B6**) Imaging of the left cheek for patient N° 37. Scale bars = 5 mm. R = right, L = left.

In addition to quantitative measurements, Antera 3D imaging provided visual representations of the skin’s texture (Figure 4).

The gathered images highlighted the decrease in skin surface texture irregularities over time, corroborating the reductions in texture score and roughness observed in the reported numerical data (Figure 3, Table S3).

### 2.4. DermaScan Results for Dermal Structure and Thickness

The variations in dermal thickness and structure were monitored using the DermaScan device. Dermal thickness was initially measured at an average of 1.75 ± 0.19 mm. After the Hydragel A1 injections, at the follow-up visit at D28, the dermal thickness significantly increased to 1.89 ± 0.21 mm (i.e., *p*-value < 0.001 Wilcoxon test; Table 1).

**Figure 5:**
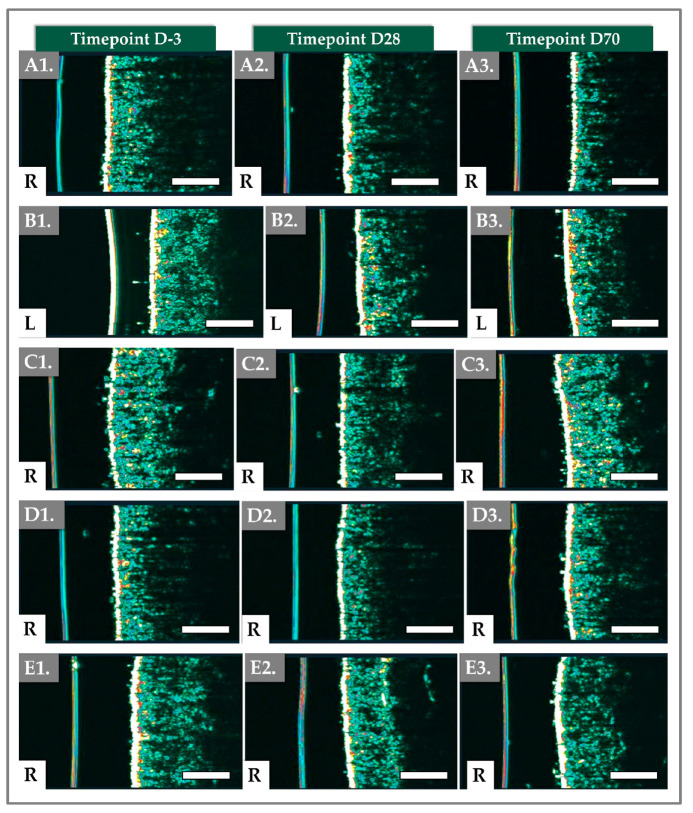
Results of DermaScan imaging at various timepoints of the clinical study. (**A1**–**A3**) Imaging of the right cheek for patient N° 15. (**B1**–**B3**) Imaging of the left cheek for patient N° 24. (**C1**–**C3**) Imaging of the right cheek for patient N° 26. (**D1**–**D3**) Imaging of the right cheek for patient N° 30. (**E1**–**E3**) Imaging of the right cheek for patient N° 43. Scale bars = 2 mm. R = right, L = left.

This result was, however, not sustained at D70, as a decrease in the skin thickness was observed (i.e., 1.70 ± 0.18 mm, *p*-value = 0.016; Paired *t*-test; Table 1). The total intensity followed a similar trend, with the mean increasing from pretreatment to post-treatment at D28 (i.e., 14.90 ± 3.20 mm vs. 13.46 ± 2.56 mm; *p*-value < 0.003; Paired *t*-test), and then decreasing to a value lower than the pre-treatment level (i.e., 13.09 ± 2.97 mm; *p*-value = 0.002; Wilcoxon test; Table 1). Examples of cross-sectional images of the skin captured by the DermaScan device are presented in Figure 5 and Figure S2, highlighting the distinct dermal layers.

The epidermis structure appeared to remain consistent throughout the study period (Figure 5).

### 2.5. Chromameter Results for Skin Tone and Color Balance

**Figure 6:**
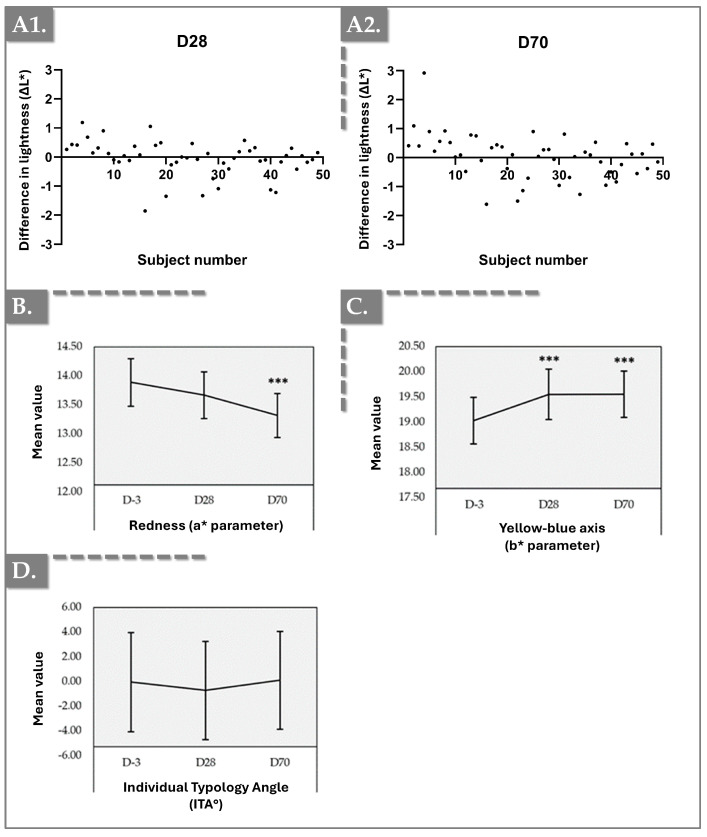
Clinical monitoring results using the Chromameter device. (**A1**,**A2**) ∆L * parameter (lightness) distribution evolution per patient between 28 days and 70 days of follow-up. (**B**) a * parameter (redness) evolution. (**C**) b * parameter (yellow–blue axis) evolution. (**D**) ITA° parameter (Individual Typology Angle) evolution. Error bars = 95% confidence interval. Statistical significance (i.e., *p*-value ≤ 0.001) was evidenced by three asterisks (***). Detailed statistical analysis results are reported in Tables S5 and S6.

The Chromameter analysis revealed significant improvements in skin tone and color balance after product administration, with the effects persisting throughout D28 and D70. Measurements of brightness (L), redness (a), and yellowness (b) parameters demonstrated meaningful changes, reflecting enhanced skin radiance and uniformity over time compared to baseline at D-3 (Figure 6 and Figure S3).

Firstly, the “∆L” parameter (difference in lightness) showed that 54% of the subjects presented a positive increase after 28 days and 64% after 70 days, confirming the improvement in skin lightness over time. Furthermore, the “a” parameter (redness) showed a reduction from 13.86 ± 1.50 at baseline to 13.26 ± 1.39 at D70, a 4.32% decrease indicating a significant reduction in skin redness. Additionally, 61% of the subjects expressed a decrease at D28 and 71% at D70. Finally, the “b” parameter (may indirectly correlate with melanin levels and represents the yellow–blue axis) increased from 18.94 ± 1.72 at D-3 to 19.50 at D28 and D70, showing a significant increase in skin yellowness. Moreover, 69% expressed and increase at D28 and 77% at D70. No significant changes are observed with the Individual Typology Angle. These results supported the clinical efficacy of Hydragel A1 in improving skin brightness and color uniformity, contributing to an overall improvement in skin radiance and appearance.

### 2.6. Cutometer Results for Skin Elasticity and Firmness

**Figure 7:**
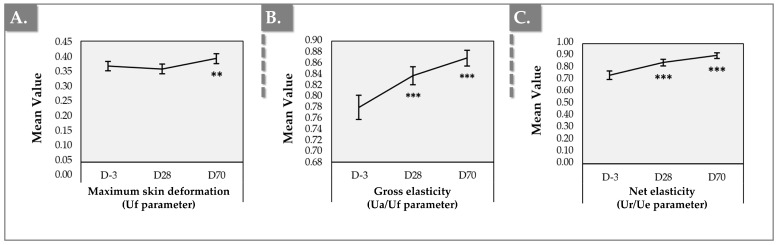
Clinical monitoring results using the Cutometer device. (**A**) “Uf” parameter (maximum skin deformation) evolution. (**B**) “Ua/Uf” parameter (gross elasticity) evolution. (**C**) “Ur/Ue” parameter (net elasticity) evolution. Error bars = 95% confidence interval. Statistical significance was evidenced by two asterisks (i.e., “**” for *p*-value ≤ 0.01) or by three asterisks (i.e., “***” for *p*-value ≤ 0.001). Detailed statistical analysis results are reported in Table S7.

The instrumental measurements using the Cutometer device demonstrated significant improvements in skin elasticity and firmness after product administration, with effects sustained throughout D70. Key sub-parameters, including maximum skin deformation (Uf), gross elasticity (Ua/Uf), and net elasticity (Ur/Ue), showed statistically significant improvements compared to baseline values at D-3 (Figure 7 and Figure S4, Table S6).

Firstly, the maximum deformation (R0 or Uf) demonstrated a slight decrease from an average of 0.36 ± 0.06 at baseline (D–3) to 0.35 ± 0.06 at Day 28, which was not statistically significant (Figure 7A, Table S6). However, a significant increase was observed at Day 70, with a measurement of 0.39 ± 0.07, representing a notable difference of 7.81% from baseline (i.e., *p*-value = 0.005; Paired *t*-test; Table S7).

Secondly, gross elasticity (R2 or Ua/Uf) exhibited a significant increase following product administration. It rose from an average of 0.78 ± 0.08 at baseline (D–3) to 0.84 ± 0.06 at Day 28 and further increased to 0.87 ± 0.05 at Day 70 (Figure 7B). Both increments from D-3 to D28 and from D-3 to D70 were statistically significant, yielding an overall increase of 11.52% from D-3 to D70 (i.e., *p*-value < 0.001; Paired *t*-test; Table S7). Additionally, after 70 days, 63% of the patients showed an increase.

Thirdly, this improvement in gross elasticity was mirrored by net elasticity (R5 or Ur/Ue), which also showed consistent enhancement after treatment, increasing from 0.74 ± 0.12 at D-3 to 0.90 ± 0.08 at D70 (Figure 7C). This change represented a significant improvement in skin elasticity (i.e., *p*-value < 0.001; Paired *t*-test; Table S7).

Finally, the immediate recovery after suction release (R7 or Ur/Uf) demonstrated a similar trend, with a consistent increase following treatment, rising from 0.58 ± 0.12 at D-3 to 0.71 ± 0.08 at D70. This corresponded to an overall improvement of 22.35% (i.e., *p*-value < 0.001; Paired *t*-test; Table S7).

### 2.7. Corneometer Results for Skin Hydration

**Figure 8:**
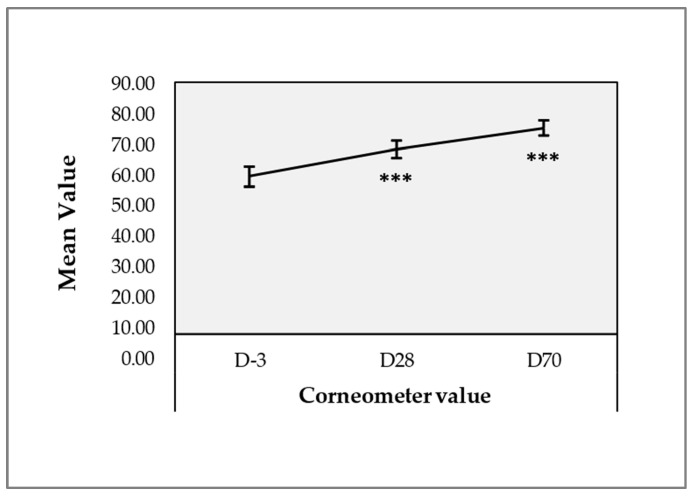
Clinical monitoring results using the Corneometer device. Error bars = 95% confidence interval. Statistical significance (i.e., *p*-value ≤ 0.001) was evidenced by three asterisks (***). Detailed statistical analysis results are reported in Table S7.

The Corneometer measurements demonstrated a marked improvement in skin hydration following product administration, with hydration levels increasing significantly at D28 and D70 compared to baseline. Importantly, key hydration parameters revealed sustained changes, highlighting the effectiveness of Hydragel A1 in improving moisture retention in the skin. The mean hydration level increased from 56.49 ± 12.45 at D-3 to 66.24 ± 10.72 at D28, reflecting a 17.26% improvement (i.e., *p*-value < 0.001; Wilcoxon signed-rank test; Figure 8, Table S8).

This improvement was sustained throughout D70, with an average hydration level of 73.87 ± 9.52, indicating a long-lasting effect on skin moisture content. Median hydration values showed a similar trend, increasing from 57.37 at baseline to 66.35 at D28 and 73.85 at D70 (Table S8). Minimum and maximum values also improved, with the minimum hydration level rising from 29.07 at D-3 to 45.88 at D28, and the maximum increasing from 83.08 at baseline to 90.57 at D28, reflecting an overall enhancement in hydration (Table S8). Additionally, 100% of the subjects showed an increase in hydration at D70.

### 2.8. Safety and Injectability Evaluation Results

The clinical safety profile of Hydragel A1 was thoroughly assessed throughout the study by monitoring for the occurrence of injection site reactions (ISRs) and adverse events (AEs). Overall, the treatment was well tolerated, with most reactions being mild and resolving within the expected timeframe (i.e., 1–4 h). Specifically, the injector’s assessment of injection tolerance in participants, based on ISR occurrence, indicated that the injections were generally well tolerated. Any reactions observed occurred on Days 0 and 14, resolving spontaneously, with no ISRs reported at Days 28 and 70. The most frequently observed reactions were light redness (i.e., 88% of patients at D0 and D14). Other reactions that were observed shortly after the injections on D0 and D14 included pain, firmness, swelling and bruises, affecting 2% to 59% of patients, mostly with mild intensity.

Therein, the most frequently observed reactions were redness (i.e., 81% of patients) and swelling (83%). Additional common reactions included pain and firmness, with the majority of all reactions reported as mild in intensity (Table 2). Importantly, no adverse events or severe adverse events associated with the interventions were reported throughout the study, further supporting the safety of Hydragel A1 as an injectable treatment. The absence of serious complications or prolonged adverse effects indicated a favorable safety profile for the product. Finally, an analysis of the ease of administration of the Hydragel A1 device was performed based on investigator-reported gradings (Table 3).

For all subjects, the injectors were very satisfied/satisfied with the ease of extraction, ease of injection and immediate results on D0 and D14. Globally, the results of the clinical study underlined that the Hydragel A1 device was safe under the prescribed conditions of use and was easily applied by the investigators.

### 2.9. General Discussion

#### 2.9.1. Combining Injectable Ingredients for Enhanced Aesthetic Function

Facial skin aging is a multifaceted process, characterized by alterations in skin elasticity, hydration, texture, and pigmentation. The thriving demand for non-invasive or minimally invasive interventions has spurred the proliferation of commercial injectable treatments designed to enhance skin quality and mitigate the effects of photoaging [10]. This clinical study evaluated the safety and efficacy of Hydragel A1, a device formulation combining hyaluronic acid (HA), niacinamide, and tranexamic acid (TXA), to improve facial skin quality. Elucidating how combined additional ingredients in injectable formulations can synergistically address multiple facets of skin aging is paramount in aesthetic dermatology. This study addresses a critical gap by assessing a single injectable product that simultaneously targets multiple aging mechanisms, thereby reducing the necessity for sequential treatments. It is important to note that the existing literature is predominantly focused on topical applications of niacinamide and TXA, often in combination, but rarely with HA. Only a few studies explore the topical co-application of all three agents, and to our knowledge, no clinical studies have evaluated this specific triple combination in an injectable format. This underscores the novelty of the use of a multi-agent injectable combining HA, niacinamide, and TXA, which represents a relatively new approach in aesthetic and regenerative medicine, aiming to simultaneously address hydration, photoaging, and elasticity within a single hydrogel platform. Therein, the incorporation of the “Boost and Shield” technology to the investigated hydrogel offers added value to the product design.

Firstly, HA is widely recognized for its capacity to hydrate the skin, enhance elasticity, and provide volumization. Its hydrophilic properties augment moisture retention, consequently plumping the skin and attenuating the appearance of wrinkles [5,11]. Evidence suggests that injectable HA may also stimulate fibroblast activity, leading to increased collagen deposition and improved dermal extracellular matrix integrity [12]. Secondly, niacinamide, a well-established derivative of vitamin B3, enhances skin barrier function, diminishes fine lines, lightens hyperpigmentation, and reduces skin roughness [13,14,15,16]. Thirdly, TXA prevents UV-induced pigmentation and hyperpigmentation disorders, such as melasma [17,18,19]. While topical applications of these molecules have gained considerable popularity, emerging evidence suggests that injectable formulations yield superior outcomes [20,21]. Indeed, injectable treatments facilitate deeper dermal penetration, directly targeting the dermis where critical processes, such as collagen synthesis and hydration alterations, occur. In contrast, topical applications must traverse the skin barrier, primarily affecting the epidermis [22,23].

It is important to note that injectable niacinamide is an option in aesthetic medicine and other applications. Niacinamide has been incorporated into injectable formulations for non-cosmetic purposes, including the treatment of vitamin deficiencies (e.g., Infuvite, FDA-approved) and as an absorption modifier in fast-acting subcutaneous insulin formulations (e.g., Fiasp, FDA- and EMA-approved). Niacinamide is also present in formulations such as Innoryos, a viscosupplementation product for osteoarthritis, demonstrating the rationale for injectable niacinamide [24]. Furthermore, injectable niacinamide has been widely adopted in cosmetic dermatology within dermal filler products and dermboosters like Innovyal Regenerative Action or NCTF 135HA [25].

Despite its small molecular size (i.e., 122.1 Da), the aqueous solubility (i.e., 212.95 mg/mL, log p value of −0.37) of niacinamide suggests that topical delivery may be suboptimal [13,23]. Thus, the integration of niacinamide into injectable HA-based gels represents a promising innovation for skin rejuvenation treatments, offering a dual-action approach by addressing skin concerns and potentially enhancing the longevity of the HA hydrogel [26]. Specifically, the antioxidant properties of niacinamide can protect HA chains from degradation by reactive oxygen species (ROS) and enzymatic breakdown by hyaluronidases, potentially extending the in vivo residence time of the HA gel [26]. This protective effect could lead to prolonged efficacy, reducing the need for frequent reinjections and offering more sustained aesthetic improvements.

Tranexamic acid, initially developed for its oral application in treating bleeding disorders, has gained prominence in dermatological applications, particularly for managing hyperpigmentation [27,28]. Recent studies have increasingly focused on the topical administration of TXA, demonstrating its potential to improve skin tone [29,30,31]. The efficacy of TXA in topical formulations has encouraged its incorporation into cosmeceutical products, reflecting a growing interest among practitioners in leveraging its skin-lightening properties. TXA is registered in the European Glossary of Common Ingredient names for the purpose of labeling cosmetic products placed on the market, as established by Decision (EU) 2019/701 of 5 April 2019—Cosing database. Additionally, tranexamic acid is recognized by the European Chemicals Agency (ECHA) for its consumer uses in products such as cosmetics and personal care items. This inclusion reflects its widespread application within the industry, particularly in products aimed at improving skin appearance.

#### 2.9.2. Focus on the Physical Properties of Hydragel A1

In clinical practice, the selection of hyaluronic acid (HA) fillers for aesthetic enhancement necessitates a thorough understanding of their rheological properties, as these characteristics dictate the filler’s behavior under various physiological conditions. Despite shared indications across different brands, HA fillers exhibit significant variability in their rheological and physicochemical properties due to formulation differences [32,33,34]. Clinicians must consider rheological parameters, such as the elastic modulus (G′), which quantifies the energy stored and recovered during shear deformation, and the viscous modulus (G″), which measures energy dissipation during deformation, as they directly influence the filler’s ability to resist shear forces and maintain structural integrity. Higher G′ values indicate firmer, more elastic fillers, specifically designed for deep-plane volume restoration. These fillers provide robust structural support with minimal migration, making them ideal for volumizing targeted areas.

Conversely, dermboosters, characterized by lower viscosity and elasticity, disperse readily within superficial soft tissue, rendering them more suitable for addressing fine lines. For instance, Hydragel A1, containing 10 mg/mL of linear HA, exhibits an elastic modulus of 1300 mPa as indicated by the supplier, enabling effective dispersion within the epidermis and dermis, which is advantageous for treating fine lines and smoothing surface irregularities. Specifically, the objective with such dermboosters is not facial contour remodeling, but rather the promotion of anti-aging effects by attenuating skin unevenness and improving texture [35,36,37].

Linear HA dermboosters typically exhibit inherently short residence times within the skin, generally ranging from 2 to 4 days, due to rapid degradation primarily mediated by endogenous hyaluronidases and reactive oxygen species (ROS), which cleave glycosidic linkages in the HA chains [36,38]. To mitigate this limitation, Hydragel A1 employs the patented Boost and Shield technology, incorporating niacinamide (i.e., at a concentration of 15 mg/mL) to protect the HA chains from oxidative degradation [26]. Additionally, Hydragel A1 includes tranexamic acid (TXA), a cosmetic ingredient. TXA is widely utilized in cosmetic formulations at concentrations up to 3.0% (30 mg/mL) for dermal application, owing to its ability to improve the skin tone. It is important to note that Hydragel A1 contains 1% (10 mg/mL) of TXA, below the 3% threshold applicable to cosmetics. At this concentration, TXA’s antioxidant and radiance activities may be attributed mainly to its structural similarity to L-lysine, a known antioxidant. As a derivative of L-lysine, TXA retains analogous functional characteristics. Its molecular structure, featuring an amine group (-NH_2_) and a carboxylic acid group (-COOH), may facilitate interactions with free radicals and inhibit oxidative processes [39,40,41]. Thus, the inclusion of niacinamide and TXA could significantly minimize the need for frequent reapplication.

#### 2.9.3. Linking Product Formulation Attributes with Clinical Results

The observed clinical outcomes can be attributed to the cumulative effects of the components within the Hydragel A1 formulation. Chromameter measurements (Figure 6) revealed an increase in the L-value (indicating skin lightness) by Day 28, which persisted through Day 70. Concurrently, the a-value (redness) demonstrated a significant decrease, and the b-value which is generally associated with melanin content or yellowness, exhibited a significant increase, collectively reflecting an amelioration of skin discoloration and irritation. A key factor contributing to these outcomes is likely the sustained release of TXA and niacinamide from the HA gel matrix. These findings are corroborated by previous clinical investigations. For instance, Saleh et al. demonstrated the efficacy of intralesional TXA injections in reducing cutaneous brown spots [42]. Similarly, another study comparing oral versus microinjected TXA in melasma patients highlighted the effectiveness of TXA injections in mitigating hyperpigmentation associated with this condition [43]. While our findings corroborated the well-established efficacy of TXA in reducing cutaneous uneven skin tone and niacinamide’s role in inhibiting melanosome transfer, our study uniquely demonstrates the amplified and sustained benefits achieved when these active ingredients are delivered within an injectable HA matrix [42,43,44,45]. This co-delivery likely facilitates the prolonged exposure of target cells to the ingredients, potentially enhancing their synergistic effects beyond what might be observed with topical applications or standalone treatments.

From an additive or synergistic perspective, niacinamide may complements TXA’s action by slowing melanosome transfer to keratinocytes, further enhancing the observed improvements in skin tone. Furthermore, niacinamide exhibits a protective effect on melanocytes against UVA and UVB radiation-induced DNA damage [44,45]. This protective mechanism suggests a potential preventive role against further cellular senescence in skin cells.

Complementary photographic evidence (Figure 2) corroborated the instrumental findings, illustrating a visible reduction in dark spots and reinforcing the efficacy of the injectable treatment in improving skin appearance. Collectively, these results underscore the synergistic roles of TXA and niacinamide in promoting a more uniform skin tone. Parallelly, the GAIS results demonstrated clear immediate and sustained improvements in skin appearance following treatment. At Day 0, 100% of participants were rated “Improved” by the injector, and by Day 28, 89.6% were classified as “Much improved” or “Very much improved”, with these results maintained through Day 70. These findings highlight both the rapid onset and durability of the aesthetic benefits.

The immediate improvements observed at Days 0 and 14 are primarily attributable to HA, renowned for its hydrophilic properties. Upon injection, HA draws water into the dermis, creating immediate hydration and volumization [46]. This process mechanically smooths fine lines and improves skin texture by expanding the dermal layer, enhancing skin plumpness, and reducing the appearance of superficial wrinkles [5].

Beyond these immediate effects, the sustained improvements observed at Days 28 and 70 are likely due to the cumulative action of HA, TXA, and niacinamide. Research indicates that injected HA stimulates dermal fibroblasts by binding to TGF-β receptors, activating a signaling pathway that promotes collagen synthesis [47,48]. This process supports the maintenance of extracellular matrix (ECM) homeostasis, which deteriorates with age, partly due to ROS progressively inhibiting TGF-β signaling [16,47]. TXA, by inhibiting ROS, mitigates UV-induced damage and the downstream effects of oxidative stress on skin health and appearance. Notably, at the concentrations used, this action is localized and does not induce systemic pharmacological effects [49,50,51,52,53]. TXA may also contribute to a more even skin tone by modulating melanocyte–keratinocyte interactions, and emerging evidence suggests its role in improving dermal structure. Specifically, TXA can enhance collagen synthesis in dermal fibroblasts, even after collagen production has been diminished by repeated UVA exposure, achieved through its ability to reduce intracellular ROS [52]. These actions help restore collagen production in fibroblasts damaged by intrinsic and extrinsic processes, such as chronic UV exposure [54].

Additionally, niacinamide has been extensively documented for its multifaceted roles in skin health, including enhancing collagen production, reducing oxidative stress, and improving barrier function, collectively contributing to dermal regeneration and resilience over time [13,14,16,24,55]. In vitro studies have shown that niacinamide increases collagen expression and suppresses the mRNA expression of matrix metalloproteinases, enzymes involved in dermal collagen degradation [55,56]. These enzymes are typically stimulated by excess ROS, leading to collagen degradation, and their activity increases with age [16]. Studies suggest that by reducing ROS production in fibroblasts, niacinamide contributes to extending fibroblast lifespan [57].

The structural improvements associated with these active ingredients likely explain the superior results obtained at Days 28 and 70. Unlike the immediate hydration provided by HA, the collagen-stimulating effects of HA, TXA, and niacinamide are more gradual, contributing to complex and lasting changes in dermal thickness and elasticity. These improvements are not merely cosmetic but involve integral dermal modifications, suggesting that the treatment effects could persist beyond the study’s observation period. Antera 3D assessments (Figure 3 and Figure 4) revealed a significant reduction in skin roughness, with a 13.47% improvement by Day 70. This improvement can be attributed to the skin-smoothing properties of HA and the antioxidant properties of TXA and niacinamide. HA’s hydrophilic nature facilitates increased dermal hydration, contributing to skin plumpness and resulting in a smoother texture and reduced fine wrinkles. These findings support the notion that Hydragel A1 improves hydration and plays a crucial role in enhancing overall skin quality.

This study revealed a significant enhancement in skin hydration, with a 17% increase by Day 28, sustained through Day 70 (i.e., over 30% increase). This enduring improvement is largely linked to HA’s capacity to attract and retain moisture within the dermis. The delayed yet sustained effects highlight HA’s prolonged activity in the dermal layers and gradual ECM remodeling. Cutometer assessments (Figure 7) indicated significant improvements in gross elasticity (R2 or Ua/Uf) and net elasticity (R5 or Ur/Ue), evident by Day 28 and sustained through Day 70. Specifically, R2 (i.e., gross elasticity/viscoelasticity, the skin’s resistance to mechanical suction versus recovery, R2 = Ua/Uf) improved by 11.52%, indicating increased overall skin firmness. Concurrently, R5 (i.e., net elasticity, skin’s ability to revert to its original state after deformation) exhibited a 22.31% increase, signifying improved collagen fiber remodeling [58]. These findings suggest that Hydragel A1 (i.e., specifically HA and niacinamide) actively stimulates fibroblasts, promoting collagen and elastin production, and alters mechanical properties due to increased water content [24,26,59]. While HA’s effects on hydration are rapid, collagen turnover is slower, resulting in peak elasticity improvements manifesting later and sustaining longer than superficial hydration.

Furthermore, the concurrent increase in L-value (lightness) and b-value (yellowness/melanin content) alongside a significant decrease in a-value (redness) provides a more comprehensive picture of skin dyschromia amelioration than is often reported for treatments targeting individual chromophores. This suggests a multi-pronged attack on various facets of discoloration, extending beyond simple melanin reduction to address overall skin tone uniformity and irritation, a critical yet often underemphasized aspect of aesthetic improvement.

The Hydragel A1 formulation of HA, TXA, and niacinamide, administered via dermal injection, demonstrated enhanced and prolonged effects on hydration, skin texture, and pigmentation. This study contributes to the growing body of the literature supporting the synergistic potential of combining HA with TXA and niacinamide for short-term and long-term skin improvement, particularly in addressing hydration, pigmentation, and structural integrity.

#### 2.9.4. Outlook on the Temporality of the Clinical Results

The study’s findings demonstrated sustained improvements in skin texture, hydration, elasticity, and pigmentation throughout the 70-day monitoring period. DermaScan ultrasound measurements indicated an increase in dermal thickness by Day 28, reflecting the volumizing and hydrating effects of HA. However, by Day 70, a diminution of these gains was observed. The decrease in skin thickness at Day 70 may be attributed to the natural degradation of HA. This reduction accounts for the decline in immediate volumizing effects, even as improvements in skin texture persisted. Although Hydragel A1 (i.e., comprising HA, TXA, and niacinamide) stimulates collagen production, this process is time-dependent, and the observed reduction in thickness might suggest that collagen synthesis had not fully compensated for HA breakdown by Day 70. While elasticity improvements are attributable to the presence of newly synthesized collagen and elastin, the initial plumping effect derived from HA’s water retention diminishes [60]. This transient effect supports the rationale for exploring maintenance injection schedules in future studies. Additionally, most skin quality injectable treatments currently available on the market recommend more intensive protocols, typically involving two to three injections during the initial “induction phase”, followed by monthly maintenance sessions.

The remarkable durability of improvements observed through Day 70, particularly in GAIS scores and instrumental measurements, distinguishes our findings from studies focusing solely on the transient effects of HA-based fillers. This sustained efficacy strongly suggests fundamental biological remodeling rather than just immediate cosmetic enhancement, driven by the continuous interplay of HA’s structural support and cell signaling capabilities with the anti-inflammatory, antioxidative, and collagen-stimulating properties of TXA and niacinamide.

Long-term enhancements in skin elasticity and hydration are ascribed to the combined actions of HA, niacinamide, and TXA, which collectively promote collagen synthesis and maintain skin barrier function. The injectable hydrogel’s viscosity, coupled with the synergistic actions of the active ingredients, provides a comprehensive approach to skin rejuvenation. Nonetheless, the biodegradable nature of HA leads to a gradual decline in its initial volumizing effects. Overall, the combination of HA for hydration and volume, TXA and niacinamide for texture, pigmentation, and skin barrier improvement offers a holistic solution to skin aging. This multifaceted approach targets several aging mechanisms, thereby reducing the need for multiple, disparate treatments.

Importantly, our results, showing significant enhancements in gross and net elasticity (R2 and R5), go beyond typical hydration-induced improvements associated with HA. The substantial increases, especially in R5 (net elasticity), point towards genuine collagen fiber remodeling and improved dermal resilience. This aligns with in vitro evidence of niacinamide’s collagen-boosting effects and TXA’s ability to restore collagen synthesis in damaged fibroblasts, suggesting that the combined formulation effectively combats the age- and photo-induced degradation of the extracellular matrix [52,55].

#### 2.9.5. Considerations on Injection Depth and Dermal Targeting

The injection depth employed in this study was a critical consideration, specifically targeting the superficial to mid-dermis. This placement is strategically chosen for several reasons. Firstly, it allows for the optimal immediate hydrophilic action of HA, drawing water into the dermal layer to provide rapid hydration, volumization, and the smoothing of fine lines [5,46]. Secondly, this depth ensures the effective delivery and sustained release of TXA and niacinamide to their primary cellular targets. Melanocytes, responsible for pigmentation, reside at the dermal–epidermal junction, and fibroblasts, key for collagen production and ECM homeostasis, are abundant throughout the dermis. Delivering these ingredients directly into this environment maximizes their local concentration and prolonged interaction with these cells, thereby promoting sustained lightening effects and dermal regeneration.

Different injection depths can significantly influence the pharmacokinetics and clinical outcomes of injectable treatments. For instance, more superficial intradermal injections might lead to prolonged product visibility or potential inflammatory responses, while deeper subdermal injections, typically used for significant volume augmentation, may not provide optimal local concentrations for addressing superficial pigmentation and skin texture. Our chosen depth aimed to strike a balance, leveraging the immediate physical benefits of HA while ensuring the combined actions of TXA and niacinamide are effectively delivered to the precise dermal layers where they can exert their long-term effects. Future investigations exploring a range of injection depths could further refine treatment protocols to achieve highly individualized and optimized results for specific patient concerns.

#### 2.9.6. Confirmed Safety and Injectability of Hydragel A1

As previously reported, the absence of serious adverse events and injection site reactions confirms the clinical safety profile of the Hydragel A1 device. These results align with the existing literature, particularly concerning TXA, which is a component least frequently encountered in aesthetic injectable products compared to HA and niacinamide. Numerous clinical trials have been conducted utilizing intradermal/intralesional and microneedling administration of TXA [30,61,62], including studies involving multiple intradermal injections of 4 mg/mL TXA with a 48-week follow-up [62].

Furthermore, administration-related parameters and ease of use by the physician significantly influence the clinical adoption of novel injectable fillers. Specifically, the viscosity of Hydragel A1 is a critical parameter that dictates its performance during injection and its behavior within soft tissues post implantation. It reflects the hydrogel’s behavior once introduced into the tissue. Notably, Hydragel A1 exhibits non-Newtonian behavior, characterized by a decrease in viscosity with increasing shear stress. This property results in high initial resistance to flow during injection; however, once the pressure on the plunger exceeds a specific threshold, the filler reaches its “shear thinning point”, facilitating a smoother injection [63].

Positive feedback from injectors regarding the ease of use of Hydragel A1 underscored the enhanced control provided, particularly in managing the volume of the dermobooster injected. The ability to accurately regulate the injected volume minimizes the risk of overcorrection or undercorrection, which can lead to complications (e.g., the Tyndall effect) or suboptimal aesthetic results. This precision is essential for tailoring treatments to individual patient needs, ultimately contributing to improved aesthetic outcomes and enhanced patient satisfaction.

### 2.10. Study Limitations and Future Perspectives

This study provides robust evidence supporting the safety and efficacy of Hydragel A1 in enhancing skin hydration, texture, elasticity, and mitigating hyperpigmentation. However, several limitations warrant consideration. Firstly, we acknowledge the absence of a placebo or active comparator group as a limitation of the current study, as it makes it difficult to rule out the contribution of placebo effects or the skin’s natural variation over time. In detail, our primary objective was to conduct an initial clinical evaluation of Hydragel A1 in a real-world aesthetic practice to establish safety and gather preliminary efficacy data; thus, no placebo group was included. To address this limitation, future research comprises a second controlled clinical study that will include a placebo group, an active comparator (commercial reference product), and a split-face design to allow within-subject comparisons and further isolate the specific effects of Hydragel A1.

Secondly, the 70-day monitoring period, while sufficient to demonstrate significant improvements, may not fully elucidate the long-term durability of the observed results. Dermal remodeling and collagen synthesis are protracted processes, necessitating extended observation periods to accurately assess the sustained effects of Hydragel A1 on fine lines, dermal thickness, and pigmentation. Of note, the 70-day follow-up was intentionally chosen to capture early and mid-term improvements in skin hydration, texture, elasticity, and pigmentation, while minimizing the influence of seasonal environmental variability on skin parameters. However, we acknowledge that many skin quality injectable treatments currently available are used in more extensive protocols, typically involving an initial “induction phase” followed by maintenance sessions. This aspect is crucial when considering long-term outcomes such as dermal matrix remodeling and collagen synthesis. Notwithstanding, when contextualizing these results within the broader literature, it is noteworthy that the significant effects observed in this study occurred within a relatively abbreviated timeframe. Further comparative research is imperative to ascertain whether Hydragel A1’s rapid efficacy aligns with or surpasses the performance of comparable treatments over similar and extended durations [64,65,66]. A longer-term clinical trial is currently in preparation, including a maintenance protocol and the evaluation of structural biomarkers such as collagen expression and dermal thickness over time.

Thirdly, the participant cohort in this study exhibited relative homogeneity in terms of age, skin phototype, and baseline skin conditions. While the observed improvements are promising, this homogeneity limits the generalizability of the findings. Notwithstanding, the rationale behind this selection was twofold: first, to reduce inter-individual variability in this initial exploratory phase and thereby enhance the sensitivity of detecting treatment-related changes, and second, to focus on a population segment most likely to seek and benefit from this type of injectable aesthetic treatment. Middle-aged women concerned with skin hydration, pigmentation, and elasticity represent a primary target group for Hydragel A1, and therefore, their inclusion in this early-phase evaluation was intentional and clinically relevant.

Future studies should have broadened inclusion criteria to incorporate a more diverse participant pool, encompassing individuals of varying ethnicities, skin types, and degrees of hyperpigmentation. Such diversity would validate the universal applicability of Hydragel A1 across broader populations. Furthermore, stratifying participants by the specific type of hyperpigmentation such as melasma, post-inflammatory hyperpigmentation, or solar lentigines could yield valuable insights into the differential efficacy of Hydragel A1. This stratification would delineate whether certain pigmentation types exhibit a more favorable response to the treatment, thereby informing personalized therapeutic strategies. Specifically, the administration protocol in future studies could then be adapted to address different patient profiles, drawing on both the intrinsic properties of the ingredients and clinical experience in order to provide personalized treatment recommendations.

The integration of injectable “multiple compounds” such as Hydragel A1 into clinical practice represents a significant advancement in aesthetic dermatology, offering a comprehensive solution for addressing multiple signs of facial aging. However, further randomized controlled trials are essential to compare Hydragel A1 against established treatments, such as hyaluronic acid-only injections or standalone topical formulations of TXA and niacinamide. These comparative studies would elucidate the additive or synergistic benefits of Hydragel A1’s combined active ingredients and delineate its relative efficacy within the landscape of rejuvenation therapies. Comparative evaluations should also extend to gold-standard treatments in both injectable and topical categories. Quantification at the cellular level, focusing on variations in efficacy by active ingredient type and concentration, would provide a granular understanding of Hydragel A1’s performance metrics.

Additional research into the mechanisms of action of TXA and niacinamide on dermal fibroblasts and melanocytes is crucial to enhance our understanding of their roles in collagen synthesis and pigmentation regulation. The observed clinical improvements suggest complex molecular interactions that warrant further investigation. To this end, conducting biopsies to measure collagen production in treated areas could provide critical data supporting the current findings. Furthermore, identifying the specific types of collagen (e.g., collagen types I, III, or IV) synthesized in response to treatment would elucidate the quality of dermal remodeling achieved by Hydragel A1.

Finally, future studies should explore the efficacy of Hydragel A1 using diverse administration modalities. For instance, a comparative analysis of results from topical application, injectable delivery, and a combination of both could reveal optimal strategies for maximizing therapeutic outcomes. Such studies would determine whether the synergistic effects observed with Hydragel A1’s active ingredients are best harnessed through specific or combined delivery methods. This multi-modality approach would provide clinicians with robust evidence to tailor treatment plans to individual patient needs and preferences.

## 3. Conclusions

This clinical study aimed to evaluate, for the first time, the efficacy of Hydragel A1, an injectable hydrogel combining HA, niacinamide, and TXA, in enhancing facial skin quality and radiance through dermal filling and redensification. Notably, 100% of treated subjects demonstrated aesthetic improvement, as assessed by the evaluator’s Global Aesthetic Improvement Scale (GAIS), four weeks post initial injection (two injections at Day 0 and Day 14). Consistent with this, both evaluator and subject assessments confirmed sustained global aesthetic improvements through Day 70.

Objective instrumental analyses corroborated these findings, demonstrating statistically significant enhancements in skin texture, roughness, firmness, elasticity, hydration, and the evenness of tone in comparison with the values before the first injection (i.e., the initial state of the skin). Specifically, Antera 3D measurements revealed consistent improvements in texture parameters, including a significant reduction in skin roughness (Ra and Rq) and maximum height from Day 0 to Day 70. Colorimetric analysis using the Chromameter showed statistically significant alterations in a* (redness) and b* (indirectly correlate with melanin) values, indicating improved skin color properties. Dermascan ultrasound measurements indicated dynamic responses in skin density and structure, with changes observed in segmented area, total intensity, and thickness, suggesting potential improvements in collagen organization and skin composition. Cutometer assessments demonstrated significant improvements in skin elasticity, particularly in gross elasticity and net elasticity, reflecting enhanced skin firmness and resilience. Corneometer measurements confirmed a significant increase in skin hydration levels, emphasizing the efficacy of Hydragel A1 in enhancing moisture retention.

The safety profile of Hydragel A1 was favorable, with transient injection site reactions, such as redness, pain/sensitivity, hardening/firmness, and swelling, primarily observed at Day 0 and Day 14 post injection, and resolving without sequelae by follow-up visits. No allergic reactions were reported throughout the study. The study’s limitations included the relatively short follow-up duration, the lack of a placebo or comparator group, the small and homogeneous sample size, and the need for longer-term efficacy data. Notwithstanding, the described rationale elements and perspectives of future research addressed most of these points. Collectively, the presented results underscore the safety and multifaceted efficacy of Hydragel A1 in improving various aspects of facial skin quality and appearance, including significant enhancements in texture, pigmentation, dermal structure, elasticity, and hydration, as evidenced by both subjective and objective assessments.

## 4. Materials and Methods

### 4.1. Clinical Study Design

This study was designed as a prospective, open-label clinical trial to evaluate the safety and efficacy of Hydragel A1 injections in the skin texture. Specifically, this study aimed to assess the product’s efficacy in enhancing aesthetic skin quality, including skin elasticity or firmness, and the facial skin radiance of the treated zone through dermal filling and redensification, and we evaluated its tolerability. Thus, this exploratory study prospectively collected safety and performance clinical data for the product. Participants served as their own baseline controls. This study was conducted over a 10-week period, from August 2023 to November 2023, at a contract research organization (CRO; CIDP Ltée, Phoenix, Mauritius). The clinical trial adhered to Good Clinical Practice (GCP) guidelines and the ethical principles outlined in the Declaration of Helsinki [67]. Approval for the clinical protocol was obtained from an Independent Ethics Committee, and written informed consent was secured from each participant prior to their inclusion in the study and for the publication of the results. In detail, Table 4 summarizes the clinical study design, including the various timepoints.

### 4.2. Patient Selection Methodology

In this clinical study evaluating Hydragel A1, rigorous inclusion and exclusion criteria were implemented to ensure participant safety and the validity of the results (Supplementary Methods). Eligible participants were males and females aged 18 to 45 years, with Fitzpatrick skin phototypes III to V, seeking enhancements in skin brightness and overall skin quality. Participants were required to be in good general and mental health, possess the cognitive capacity to comprehend the study’s objectives, and provide informed consent. They were mandated to refrain from other facial aesthetic procedures during the study and to adhere to the prescribed study schedule and follow-up visits. Exclusion criteria encompassed any systemic diseases or dermatological disorders that could confound the study results, known hypersensitivity to any formulation components, severe allergies, autoimmune diseases, and bleeding disorders. Participants with active cutaneous infections, those undergoing anticoagulant therapy, pregnant or breastfeeding women, and those unable to comply with study instructions or under legal restrictions were also excluded.

Ultimately, a cohort of 49 female participants, aged 20 to 45 years (i.e., mean age 33 ± 1 years), with Fitzpatrick skin phototypes ranging from III to V (i.e., 14% type III, 45% type IV, and 41% type V), were enrolled in the study. The general health status of all participants was deemed ‘normal’, as were their baseline skin conditions. The analyses were performed on all 49 included subjects (i.e., except for D28 analyses, which were performed on 48 subjects). They completed the study without any major protocol deviation (i.e., except for subject CIDP-MRU-0023, who missed visit 4).

### 4.3. Investigational Test Item

The Hydragel A1 device is a sterile, transparent and resorbing gel of HA with TXA and niacinamide. This injectable gel is packaged in sterile and single-use 6 mL glass vials, with a fill volume of 3.3 mL. The product composition is detailed in Table 5.

The Hydragel A1 product was stored at controlled temperatures between 2 °C and 25 °C throughout the study. Hydragel A1 is classified as a Class III medical device, according to Rule 8, Chapter III of Regulation (EU) 2017/745. The product is sterilized via moist heat and consists of a resorbable hyaluronic acid (HA) gel of biofermentative origin. TXA, a substance widely utilized in the cosmetic industry, is incorporated into the gel to enhance skin radiance. By filling and redensifying the dermal tissue, addressing age-related skin thinning and fragility, Hydragel A1 aims to improve skin quality, including skin elasticity and firmness. Consequently, Hydragel A1 is designed to prevent and counteract the skin aging process while promoting tissue remodeling with a corrective effect.

The osmolality of Hydragel A1 was measured at 320 mOsmol/kg, indicating appropriate isotonicity with cellular fluids [68,69]. The pH was maintained at 7.4 using phosphate buffer, aligning with the physiological pH of the skin. The gel presented a clear, particle-free appearance. The elastic modulus (G′), reflecting the gel’s elastic response under stress, was determined to be 1300 mPa. The viscous modulus (G″), indicating the gel’s liquid-like behavior, was measured at 6000 mPa. Together, these elastic and viscous moduli reflect the gel’s balanced structural characteristics. Additionally, the complex viscosity, assessing overall gel thickness and ease of application, was recorded at 990 mPa·s, ensuring a smooth application.

### 4.4. Administration Protocol and Injection Technique

The intervention was performed by a healthcare professional with extensive knowledge of facial anatomy. All interventions were conducted by the same physician throughout the study to ensure consistency. The application sites were located on both cheeks, as illustrated in Figure S1. The injection area was disinfected using an antiseptic solution (Diaseptyl) prior to administration. The contents of the Hydragel A1 vial were aseptically extracted using an 18G needle attached to a 3 mL syringe. The 18G needle was subsequently replaced with a sterile 30G or 32G needle, suitable for intradermal injection. The Hydragel A1 gel was then slowly administered into the dermis. Topical anesthesia with Emla cream was provided upon participant request. The dosage protocol permitted a maximum of 3 mL per side of the face, with a total of up to 6 mL for the entire face per subject. The injected volume was determined by the injector for each intervention. The mean quantity of product injected per visit on either the right or left cheek of subjects was 1.2 mL ± 0.2 mL, with a maximum injected volume of 1.8 mL and a minimum of 0.8 mL. The initial injection was administered on Day 0, followed by the second injection two weeks later (Day 14).

### 4.5. Outcome Endpoints

Clinical monitoring was conducted to ensure that the rights and well-being of the human subjects were protected, that the conduct of the trial was in compliance with the approved protocol, ICH GCP Guidelines, and applicable regulatory requirements, and that the reported trial data were accurate, complete, and verifiable.

#### 4.5.1. Efficacy Endpoints

The primary objective of this clinical trial was to assess the clinical efficacy of two Hydragel A1 injections administered two weeks apart. The primary efficacy endpoint was the overall aesthetic improvement, as determined by the investigator and study subject using the Investigator Global Aesthetic Improvement Scale (IGAIS) and the Subject Global Aesthetic Improvement Scale (SGAIS), respectively. Both the IGAIS and SGAIS employed a 5-point ordinal rating scale (i.e., 1 = Very much improved, 2 = Much improved, 3 = Improved, 4 = No change, 5 = Worse; Table S1). IGAIS and SGAIS evaluations were conducted at four key timepoints, (i) immediately following the first injection (Day 0), (ii) immediately following the second injection (Day 14), and (iii) during follow-up visits at Days 28 and 70, to monitor both immediate and sustained changes.

Secondary efficacy endpoints included quantitative assessments of various skin parameters using dermatological instruments. These evaluations were performed at baseline (i.e., Day 3, three days prior to the first injection) and during follow-up visits at 28 and 70 days post initial injection. The specific parameters assessed and their respective instruments were as follows:

- Skin roughness and texture: Antera 3D device (Miravex, Dublin, Ireland).
- Skin density: Dermascan C USB Ultrasound System (Cortex, Aalborg, Denmark).
- Skin brightness: Chromameter CR400 instrument (Konica Minolta, Tokyo, Japan).
- Skin firmness and elasticity: Cutometer^®^ dual MPA 580 instrument (Courage + Khazaka electronic GmbH, Köln, Germany).
- Skin hydration: Corneometer CM 825 instrument (Courage + Khazaka electronic GmbH, Köln, Germany).
- Macroscopic skin images: DERMLITE lens on a Dermlite Foto Pro II, mounted on a DSLR camera, capturing small areas on both cheeks.

Detailed descriptions of the instrumental methodologies are provided in the Supplementary Methods.

#### 4.5.2. Safety and Tolerability Endpoints

Safety and tolerability were evaluated by monitoring injection site reactions (ISRs) and adverse events (AEs) throughout the study. ISRs were documented by the investigator using a 4-point ordinal scale (i.e., 1 = None, 2 = Mild, 3 = Moderate, and 4 = Severe) assessing the following reactions at the injection sites: erythema, pain/tenderness, induration/firmness, edema, lumps/bumps, ecchymosis, and pruritus. ISRs were documented by the investigator immediately following the first and second injections on Days 0 and 14, and during follow-up visits on Days 28 and 70. Additionally, participants self-reported ISRs experienced on Day 0, providing a subjective assessment of safety. All adverse events (AEs), including serious adverse events (SAEs), occurring from the initial injection on Day 0 until the final visit on Day 70, were systematically recorded.

#### 4.5.3. Injector’s Assessment of Ease of Product Use

The ease of use of the Hydragel A1 product was also evaluated by the injector using a subjective evaluation questionnaire. The questionnaire was filled following each injection on Days 0 and 14.

### 4.6. Statistical Analyses and Data Presentation

The experimental data are presented as mean values accompanied by their corresponding standard deviations, which are depicted as error bars in the graphical representations. Primary and secondary outcomes were analyzed on an intention-to-treat basis. Quantitative variables are summarized using descriptive statistics, including minimum, maximum, mean, median, and standard deviation, while qualitative variables are expressed as frequencies and percentages. For Global Aesthetic Improvement Scale (GAIS) evaluations, frequencies and percentages are presented for the five-level ordinal score. A derived binary parameter (i.e., 1 = Improvement [Very much improved, Much improved, and Improved], 2 = No change or worsening) was analyzed using a binomial exact test against a 40% benchmark at each timepoint. For the GAIS evaluations by both subject and investigator, frequencies and percentages are presented for the overall facial score. A derived binary parameter, based on the subject and investigator GAIS scores, was defined as 1 = Improved (Very much improved, Much improved, and Improved) and 0 = Not improved (No change or Worsening). At each timepoint, these parameters are described using frequencies, percentages, and 95% confidence intervals (CIs). Specifically, the null hypothesis stated that less than 40% of subjects are responders according to the GAIS. The alternative hypothesis assumed that 65% of subjects are responders. Using a two-sided exact one-sample binomial test, a sample size of 40 subjects was required to demonstrate a significant result with 90% power (α = 5%).

Injection site reactions (ISRs) were similarly summarized using frequencies and percentages, with both the four-level ordinal scale and a derived binary parameter (i.e., 1 = Presence, 0 = Absence) analyzed. For instrumental measurements, the percentage change from baseline (Day–3) was calculated using Formula (1):

(1) $$\%\;Change=\frac{Tx-T\;reference\;timepoint}{T\;reference\;timepoint}\cdot 100$$

Timepoint comparisons were assessed using either Student’s Paired *t*-test or a Wilcoxon Signed Rank Test, depending on normality as tested by the Shapiro–Wilk test at 1% significance. The null and alternative hypotheses are defined as follows:

- H0: There is no difference between the two timepoints compared.
- H1: There is a difference between the two timepoints compared.

Detailed levels of statistical significance may be found in the Results Section and in the Supplementary Tables. Statistical tests were conducted using SPSS 19.0 and Microsoft Excel (Microsoft Corporation, Redmond, WA, USA) and GraphPad Prism v.8.0.2 (GraphPad Software, San Diego, CA, USA).

## Figures and Tables

**Table 1:** Results of DermaScan analyses of dermal thickness and structure during the study. Detailed results and statistical analyses are presented in Table S4.

Parameter	D–3	D28	*p*-Value	D70	*p*-Value
Total intensity (%)	14.90 ± 3.20	13.46 ± 2.56	0.003 (Paired *t*-test)	13.09 ± 2.97	0.002 (Wilcoxon)
Thickness of epidermis and dermis (mm)	1.75 ± 0.19	1.89 ± 0.21	<0.001 (Wilcoxon)	1.70 ± 0.18	0.016 (Paired *t*-test)

**Table 2:** Detailed counts and percentages (i.e., n [%]) of subjects presenting ISRs, as evaluated by the clinical investigator. ISR, injection site reaction.

Parameter	Face Side	Timepoint	0: None	1: Light	2: Moderate	3: Severe	Absence (0)	Presence (1, 2, 3)
Redness	Right	D0 (after injection)	6 (12.2%)	43 (87.8%)	0 (0.0%)	0 (0.0%)	6 (12.2%)	43 (87.8%)
		D14	6 (12.2%)	43 (87.8%)	0 (0.0%)	0 (0.0%)	6 (12.2%)	43 (87.8%)
		D28	48 (100.0%)	0 (0.0%)	0 (0.0%)	0 (0.0%)	48 (100.0%)	0 (0.0%)
		D70	49 (100.0%)	0 (0.0%)	0 (0.0%)	0 (0.0%)	49 (100.0%)	0 (0.0%)
	Left	D0 (after injection)	6 (12.2%)	43 (87.8%)	0 (0.0%)	0 (0.0%)	6 (12.2%)	43 (87.8%)
		D14	6 (12.2%)	43 (87.8%)	0 (0.0%)	0 (0.0%)	6 (12.2%)	43 (87.8%)
		D28	48 (100.0%)	0 (0.0%)	0 (0.0%)	0 (0.0%)	48 (100.0%)	0 (0.0%)
		D70	49 (100.0%)	0 (0.0%)	0 (0.0%)	0 (0.0%)	49 (100.0%)	0 (0.0%)
Pain/Sensitivity	Right	D0 (after injection)	22 (44.9%)	24 (49.0%)	3 (6.1%)	0 (0.0%)	22 (44.9%)	27 (55.1%)
		D14	21 (42.9%)	27 (55.1%)	1 (2.0%)	0 (0.0%)	21 (42.9%)	28 (57.1%)
		D28	48 (100.0%)	0 (0.0%)	0 (0.0%)	0 (0.0%)	48 (100.0%)	0 (0.0%)
		D70	49 (100.0%)	0 (0.0%)	0 (0.0%)	0 (0.0%)	49 (100.0%)	0 (0.0%)
	Left	D0 (after injection)	22 (44.9%)	24 (49.0%)	3 (6.1%)	0 (0.0%)	22 (44.9%)	27 (55.1%)
		D14	21 (42.9%)	27 (55.1%)	1 (2.0%)	0 (0.0%)	21 (42.9%)	28 (57.1%)
		D28	48 (100.0%)	0 (0.0%)	0 (0.0%)	0 (0.0%)	48 (100.0%)	0 (0.0%)
		D70	49 (100.0%)	0 (0.0%)	0 (0.0%)	0 (0.0%)	49 (100.0%)	0 (0.0%)
Hardening/Firmness	Right	D0 (after injection)	31 (63.3%)	17 (34.7%)	1 (2.0%)	0 (0.0%)	31 (63.3%)	18 (36.7%)
		D14	25 (51.0%)	24 (49.0%)	0 (0.0%)	0 (0.0%)	25 (51.0%)	24 (49.0%)
		D28	48 (100.0%)	0 (0.0%)	0 (0.0%)	0 (0.0%)	48 (100.0%)	0 (0.0%)
		D70	49 (100.0%)	0 (0.0%)	0 (0.0%)	0 (0.0%)	49 (100.0%)	0 (0.0%)
	Left	D0 (after injection)	31 (63.3%)	17 (34.7%)	1 (2.0%)	0 (0.0%)	31 (63.3%)	18 (36.7%)
		D14	25 (51.0%)	24 (49.0%)	0 (0.0%)	0 (0.0%)	25 (51.0%)	24 (49.0%)
		D28	48 (100.0%)	0 (0.0%)	0 (0.0%)	0 (0.0%)	48 (100.0%)	0 (0.0%)
		D70	49 (100.0%)	0 (0.0%)	0 (0.0%)	0 (0.0%)	49 (100.0%)	0 (0.0%)
Swelling	Right	D0 (after injection)	32 (65.3%)	17 (34.7%)	0 (0.0%)	0 (0.0%)	32 (65.3%)	17 (34.7%)
		D14	20 (40.8%)	29 (59.2%)	0 (0.0%)	0 (0.0%)	20 (40.8%)	29 (59.2%)
		D28	48 (100.0%)	0 (0.0%)	0 (0.0%)	0 (0.0%)	48 (100.0%)	0 (0.0%)
		D70	49 (100.0%)	0 (0.0%)	0 (0.0%)	0 (0.0%)	49 (100.0%)	0 (0.0%)
	Left	D0 (after injection)	32 (65.3%)	17 (34.7%)	0 (0.0%)	0 (0.0%)	32 (65.3%)	17 (34.7%)
		D14	20 (40.8%)	29 (59.2%)	0 (0.0%)	0 (0.0%)	20 (40.8%)	29 (59.2%)
		D28	48 (100.0%)	0 (0.0%)	0 (0.0%)	0 (0.0%)	48 (100.0%)	0 (0.0%)
		D70	49 (100.0%)	0 (0.0%)	0 (0.0%)	0 (0.0%)	49 (100.0%)	0 (0.0%)
Bruising	Right	D0 (after injection)	48 (98.0%)	1 (2.0%)	0 (0.0%)	0 (0.0%)	48 (98.0%)	1 (2.0%)
		D14	48 (98.0%)	1 (2.0%)	0 (0.0%)	0 (0.0%)	48 (98.0%)	1 (2.0%)
		D28	48 (100.0%)	0 (0.0%)	0 (0.0%)	0 (0.0%)	48 (100.0%)	0 (0.0%)
		D70	49 (100.0%)	0 (0.0%)	0 (0.0%)	0 (0.0%)	49 (100.0%)	0 (0.0%)
	Left	D0 (after injection)	49 (100.0%)	0 (0.0%)	0 (0.0%)	0 (0.0%)	49 (100.0%)	0 (0.0%)
		D14	49 (100.0%)	0 (0.0%)	0 (0.0%)	0 (0.0%)	49 (100.0%)	0 (0.0%)
		D28	48 (100.0%)	0 (0.0%)	0 (0.0%)	0 (0.0%)	48 (100.0%)	0 (0.0%)
		D70	49 (100.0%)	0 (0.0%)	0 (0.0%)	0 (0.0%)	49 (100.0%)	0 (0.0%)

**Table 3:** Count and percentage (i.e., n [%]) of subject cases for ease of product administration based on the injector’s assessments.

Parameter	Timepoint	Very Satisfied	Satisfied	Neither Satisfied nor Dissatisfied	Dissatisfied	Very Dissatisfied
Ease of extraction	D0 (after injection)	33 (67.3%)	16 (32.7%)	0 (0.0%)	0 (0.0%)	0 (0.0%)
	D14	40 (81.6%)	9 (18.4%)	0 (0.0%)	0 (0.0%)	0 (0.0%)
Ease of injection	D0 (after injection)	41 (83.7%)	8 (16.3%)	0 (0.0%)	0 (0.0%)	0 (0.0%)
	D14	35 (71.4%)	14 (28.6%)	0 (0.0%)	0 (0.0%)	0 (0.0%)
Immediate result	D0 (after injection)	9 (18.4%)	40 (81.6%)	0 (0.0%)	0 (0.0%)	0 (0.0%)
	D14	9 (18.4%)	40 (81.6%)	0 (0.0%)	0 (0.0%)	0 (0.0%)

**Table 4:** Overview of the clinical study design. AE, adverse event; IGAIS, Investigator Global Aesthetic Improvement Scale; ISR, injection site reaction; SAE, serious adverse event; SGAIS, Subject Global Aesthetic Improvement Scale.

Study Phase/Activities	D-30	D-3	D0	D14	D28	D70
Participant inclusion	Yes	/	/	/	/	/
Medical examination	Yes	/	/	/	/	/
Hydragel A1 injection	/	/	Yes	Yes	/	/
IGAIS	/	/	Yes	Yes	Yes	Yes
SGAIS	/	/	Yes	Yes	Yes	Yes
ISR by the injector	/	/	Yes	Yes	Yes	Yes
Photographs of the cheek area using Dermlite	/	Yes	/	/	Yes	Yes
Antera 3D measurement	/	Yes	/	/	Yes	Yes
Cutometer measurement	/	Yes	/	/	Yes	Yes
DermaScan measurement	/	Yes	/	/	Yes	Yes
Corneometer measurement	/	Yes	/	/	Yes	Yes
Chromameter measurement	/	Yes	/	/	Yes	Yes
Recording of AE and SAE	/	Yes	/	Yes	Yes	Yes

**Table 5:** Key active ingredients of the investigated Hydragel A1 ^1^ formulation and their percentages.

Key Ingredient	Percentage in Finished Product
Hyaluronic acid	1.00%
Tranexamic acid	1.00%
Niacinamide	1.50%

^1^ Hydragel A1 is a medical device intended to be wholly resorbed over time, which progressively reduces the space-filling effect of the product at the injection site. The kinetics of absorption of the viscoelastic gel depends on several factors, among which are the injected quantity, the depth of injection, and the patient metabolism. The expected lifetime of the product is up to 1 month after administration in the skin tissues of the patient.

## Data Availability

The original contributions presented in this study are included in the article/Supplementary Materials. Further inquiries can be directed to the corresponding author.

## Supplementary Materials

The following supporting information can be downloaded at https://www.mdpi.com/article/10.3390/gels11070495/s1, Supplementary Methods 1.1. to 1.8.: Patient inclusion/exclusion criteria and description of the function and applied methodology of the instrumental equipment used in the clinical study [70,71,72,73,74,75,76,77,78,79,80,81,82,83]; Figure S1: Zones of the facial skin that were targeted for product administration; Figure S2: Cross-sectional image of the skin obtained by DermaScan imaging. The epidermis appears as a white band, the dermis structures appear in yellow and red, and the subcutaneous tissue layer appears in green and black; Figure S3: A visual representation of the CIE Lab color space system. Reproduced from [77]; Figure S4: Visual representation of the parameters measured by the Cutometer instrument during facial skin elasticity assessments. Adapted from [58]; Table S1: A description of the significance levels for global aesthetic scores; Table S2: Count and percentage (n[%]) of subjects for GAIS scores, by evaluator and category. Statistical analysis was performed with a binomial test of proportion versus 0.4. GAIS, global aesthetic improvement scale; Table S3: Descriptive statistics and evolution over time for skin texture parameters analyzed with the Antera 3D device. When compared to D–3, a significant improvement was noted for all parameters of interest derived from image processing, characterizing texture and roughness. The improvements ranged from 10 to 18% on D28 and 7 to 13% on D70, corresponding to the ‘max height (mm)’ and ‘texture score’, respectively; Table S4: Descriptive statistics and evolution over time for skin texture parameters analyzed with the DermaScan device. An interesting (i.e., but not statistically supported) reduction in the ‘segmented area’ was noted at D28, the timepoint beyond which the mean value of the parameter remained fairly stable. ‘Total intensity %’ was found to be significantly reduced both at D28 and D70. ‘Thickness’ was found to be significantly higher at D28, but beyond that timepoint, the mean values returned to the initial state; Table S5: “L*” parameter (lightness) values for each subject; Table S6: Descriptive statistics and evolution over time for skin texture parameters analyzed with the Chromameter device. After D28 and D70, the skin appeared less red (i.e., lower a* values), with a more pronounced yellow component (i.e., higher b* values). Fairness (ITA°) remained rather stable. The general reduction (i.e., significant at D70) noted in terms of the a* parameter (i.e., skin redness), coupled with the increase in b* (i.e., yellow component) corroborate very well with improved skin fairness following the injections. Drawing from the literature, fairer subjects generally had lower levels of a* (redness), while among the darker-skinned subjects, b* was lower; Table S7: Descriptive statistics and evolution over time for skin texture parameters analyzed with the Cutometer device. A general significant increase was noted over time for all parameters assessed (i.e., whether absolute or relative). The change was more important for the relative parameters (e.g., Ua/Uf, Ur/Ue, and Ur/Uf), which is suggestive of improved skin features characterizing firmness and elasticity; Table S8: Descriptive statistics and evolution over time for skin texture parameters analyzed with the Corneometer device. A significant increase in the hydration level was observed at all timepoints when compared to D–3.

## Abbreviations

AE	adverse event
CRO	contract research organization
DNA	deoxyribonucleic acid
ECM	extracellular matrix
EMA	European Medicines Agency
FDA	Food and Drug Administration
G′	storage modulus
G″	loss modulus
GAIS	Global Aesthetic Improvement Scale
GCP	good clinical practices
HA	hyaluronic acid
IGAIS	Investigator Global Aesthetic Improvement Scale
ISR	injection site reactions
ITA	individual typological angle
LED	light-emitting diode
mRNA	messenger ribonucleic acid
NA	non-applicable
ns	non-significant
Pa	Pascals
Pa·s	Pascal seconds
PGE_2_	prostaglandin E_2_
ROS	reactive oxygen species
s	second
SAE	serious adverse event
SGAIS	Subject Global Aesthetic Improvement Scale
TGF	transforming growth factor
TXA	tranexamic acid
USA	United States of America
UV	ultraviolet
UVA	ultraviolet A
UVB	ultraviolet B
