# Supplementary material for: Clinical Safety and Efficacy of Hyaluronic Acid–Niacinamide–Tranexamic Acid Injectable Hydrogel for Multifactorial Facial Skin Quality Enhancement with Dark Skin Lightening
Source: Gels. 2025 Jun 26;11(7):495. doi: 10.3390/gels11070495 (PMC12294899; doi:10.3390/gels11070495)
Supplement: Supplementary file 1 [file gels-11-00495-s001.zip › gels-3694001-supplementary.pdf]

gels

MDPI

Supplementary Materials:

Clinical Safety and Efficacy of a Hyaluronic Acid-Niacinamide-Tranexamic Acid Injectable Hydrogel for Multifactorial Facial Skin Quality Enhancement with Dark Skin Lightening

Sarah Hsin, Kelly Lourenço, Alexandre Porcello, Michèle Chemali, Cíntia Marques, Wassim Raffoul, Marco Cerrano, Lee Ann Applegate and Alexis E. Laurent *

1. Supplementary Methods

1.1. Patient Inclusion Criteria for the Clinical Study

To be enrolled in the clinical study, the following patient inclusion criteria were applied:

- Female and male subjects.
- Fitzpatrick skin of the face from grade III to grade V.
- Aged between 18 and 45 years old.
- Subjects seeking an improvement of their skin brightness.
- Subjects seeking an improvement of their skin quality.
- Subjects of any phototype and ethnicity.
- Subjects who have given their consent for photographs for illustration purposes.
- Subjects willing to abstain from other facial aesthetic procedures in the mid-face through the entire study duration.
- Subjects in good general and mental health in the opinion of the investigator.
- Subjects who have the ability to read and fully understand the aims of the study and its conduct and have given their free, informed and expressed written consent.
- Subjects agreeing to cooperate, in full awareness of the study objectives, and understand the necessity and the duration of the follow-up controls at the trial site to ensure perfect adherence to protocol.
- Subjects who, in the judgment of the investigator, are likely to be compliant during the study.
- Subjects willing and capable of following the study rules and a fixed schedule.
- Subjects willing to and capable of signing an informed consent document (including understanding the language).

1.2. Patient Non-Inclusion Criteria for the Clinical Study

The subjects should not have presented any of the following criteria:

- Subject with any systemic disorder or skin disease that would in any way confound interpretation of the study results.
- Subjects with a medical/surgical/severe allergy/anaphylactic shock history that, in the opinion of the investigator, could compromise the safety of the subject or affect the outcome of the study.
- Subjects with a known risk of hypersensitivity to one of the components of the composition.
- Subjects suffering from autoimmune disease.
- Subjects with cutaneous disorders, inflammation or infection (e.g., herpes, acne, etc.) at the treatment site or nearby.
- Subjects for whom their medical history shows a degree of sensitivity that could lead to a reaction to the treatment, Subjects with bleeding disorders or subjects who are undergoing treatment with thrombolytics or anticoagulants.

- Subjects with a tendency to form keloids, hypertrophic scars or any other healing disorders.
- Subjects who are currently following a skin treatment.
- Pregnant or breastfeeding women or those considering a pregnancy during the study.
- Female subjects of childbearing potential with a positive urine pregnancy test at D–3 to D0.
- Subjects who have been deprived of their freedom by administrative or legal decision or who are under guardianship.
- Subjects who cannot be contacted by telephone in case of an emergency.
- Subjects in an exclusion period or participating in another biomedical research study (self-reported).
- Intellectual/mental inability to follow study instructions (if suspected) or incapacitation.

1.3. Antera 3D Device: Rationale and Applied Method

Skin texture and topography were assessed using the Antera 3D system, a non-invasive imaging device designed to capture and analyze skin surface characteristics. For each participant, high-resolution images of a 56 mm × 56 mm skin area were acquired from predetermined facial areas on both cheeks (Figure S1).

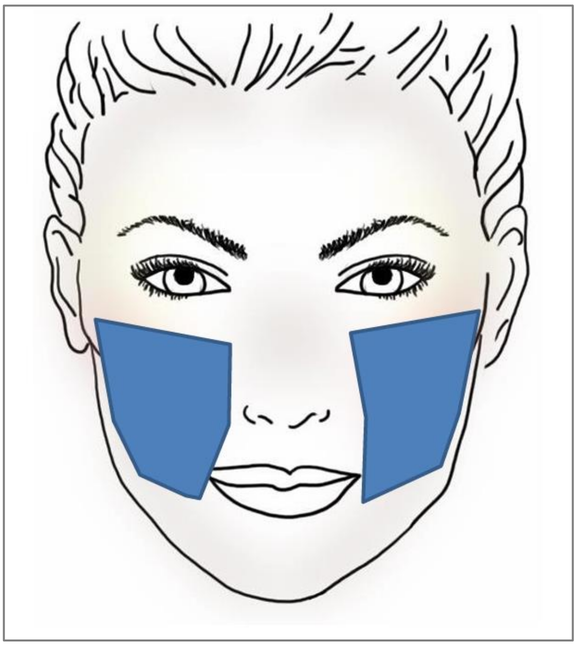

Figure S1. Zones of the facial skin that were targeted for product administration.

The image was captured in triplicate and the camera was positioned perpendicular to the skin surface, with the imaging window placed in direct contact with the skin to minimize interference from ambient lighting. The Antera 3D system employs a multi-spectral imaging approach, utilizing light-emitting diodes (LEDs) that emit light at various wavelengths and angles to capture detailed topographical images of a defined skin area [70]. The device's proprietary software reconstructs the captured images into a three-dimensional topographical map, providing quantitative data on skin texture metrics chromophore distribution [71,72]. For this study, only texture parameters were analyzed, including Ra (i.e., arithmetical mean roughness) to measure overall surface roughness, Rq (i.e., root mean square roughness) to highlight more significant deviations from the surface, and Rp (i.e., maximum height) to assess the prominence of peaks and depressions. The system calculated a texture score, an integrated measure providing a comprehensive assessment of the overall skin texture [73]. In addition to these quantitative metrics, the

Antera 3D system provides visual images highlighting the surface roughness, as shown in Figure 4.

1.4. Dermascan C USB Ultrasound System: Rationale and Applied Method

Skin density and structural changes were evaluated using the DermaScan C USB Ultrasound System, a high-frequency ultrasound scanner. DermaScan C uses a 20 MHz high-frequency ultrasound device that captures soft tissue high-resolution images [74]. A transducer was positioned on designated areas of the cheeks following the application of an ultrasonographic gel. The measurement generated a cross-sectional image of the skin up to a depth of 2.5 cm [75]. The device works by sending ultrasonic waves that are partially reflected by cutaneous structures, producing echoes with different amplitudes. The reflected echoes are converted into a two-dimensional colored image showing three structural layers: i) the epidermis, ii) the dermis, and iii) the subcutaneous tissue, as shown in Figure S2 [76].

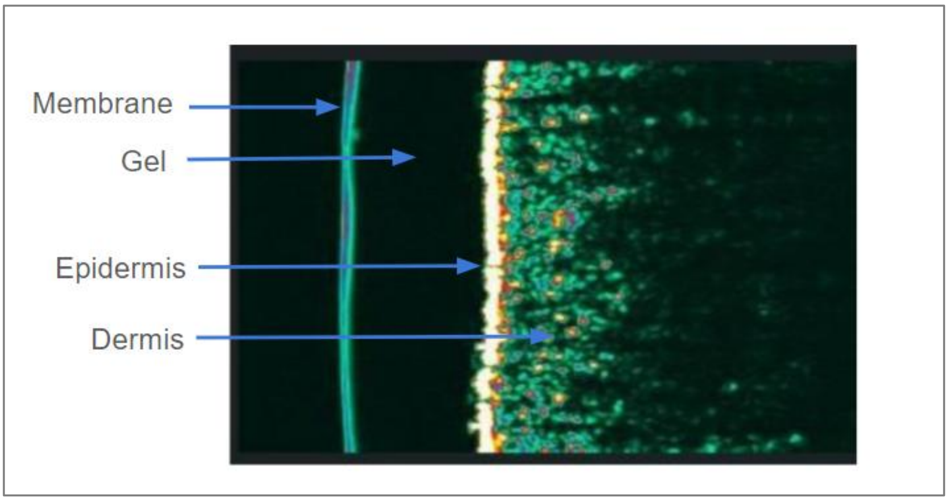

Figure S2. Cross-sectional image of the skin obtained by DermaScan imaging. The epidermis appears as a white band, the dermis structures appear in yellow and red, and the subcutaneous tissue layer appears in green and black.

The device, coupled with an image processing software, calculates two key parameters. Total intensity (%) reflects the amplitude of the reflected ultrasound waves, which correlates with tissue density. Higher total intensity values indicate denser or more reflective tissues. The total thickness of the dermis and epidermis was measured by analyzing the distance (mm) between the epidermal surface and the lower limit of the dermal layer. These measurements provide an evaluation of dermal thickening or thinning following treatment and the impact on cutaneous structures.

1.5. Chromameter CR400: Rationale and Applied Method

Skin brightness and color were assessed using the Chromameter, a device used in dermatological research for quantifying skin color based on the CIE Lab color space system (Figure S3).

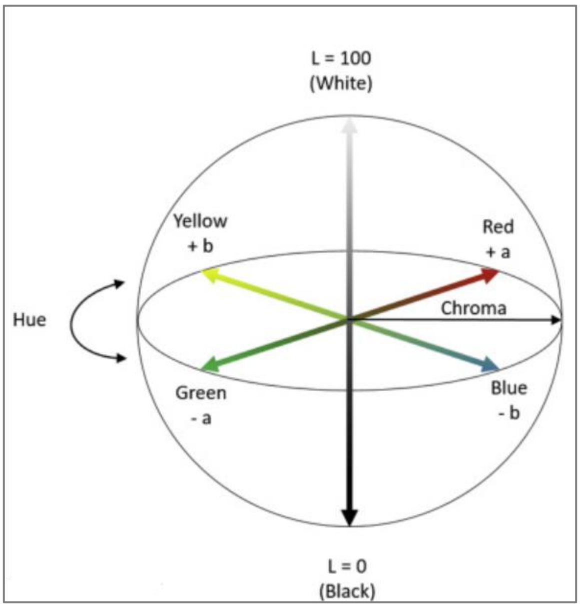

Figure S3. A visual representation of the CIE Lab color space system. Reproduced from [77].

This system provides an accurate representation of color perception with three key parameters, as listed hereafter:

- The "L*" parameter represents the luminance or lightness of the skin, with values ranging from 0 to 100, where 0 corresponds to pure black and 100 to pure white. Higher L* values indicate brighter skin.
- The "a*" parameter quantifies the red–green axis of the skin color. Positive a* values indicate a shift towards red, while negative a* values reflect a shift towards green.
- The "b*" parameter reflects the yellow–blue axis, where positive b* values correspond to increased yellowness, and negative values indicate a shift towards blue.

Furthermore, the individual typological angle (ITA°) was calculated to provide a comprehensive measure of skin brightness and pigmentation. The ITA° value is a mathematical derivation based on the L* and b* values, designed to characterize the degree of skin pigmentation by integrating both lightness and chromaticity into a single parameter. The ITA° was calculated using the following formula (Formula S1):

Formula S1 $$ITA° = \frac{180}{\pi} \cdot \left[ArcTan\left(\frac{L* - 50}{b*}\right)\right]$$

This angle provides a more comprehensive measure of skin tone, with higher ITA° values indicating lighter skin tones and lower values corresponding to darker pigmentation. The instrument was calibrated daily on a white standard plate before use to ensure accurate and consistent data collection. Each measurement consisted of three monoflash readings on a predefined area of the cheeks, with the measuring head repositioned slightly between each flash to ensure consistency. The Chromameter instrument was applied gently to the skin, avoiding excess pressure, and measurements were recorded using the MONADERM software. This method allowed for precise and repeatable assessments of skin brightness and color changes over the course of the study.

1.6. Cutometer Dual MPA 580: Rationale and Applied Method

Skin elasticity was assessed using the Cutometer Dual MPA 580, a device specifically designed to measure skin deformation in response to controlled mechanical suction. The cutometer operates by applying a controlled vacuum through a 2 mm probe to create a negative pressure of 200 mbar on the skin's surface. The skin is drawn into the probe's

aperture and the extent of deformation under suction is measured, followed by its recovery during the release phase. This method allows for the quantification of various parameters related to the skin's mechanical properties, including elasticity, firmness, and viscoelastic recovery [58]. In detail, the Cutometer Dual MPA 580 records several key parameters that reflect different aspects of skin elasticity and firmness. These parameters are derived from the skin's response to suction and its recovery. The following parameters were included in the analysis (Figure S4) [78].

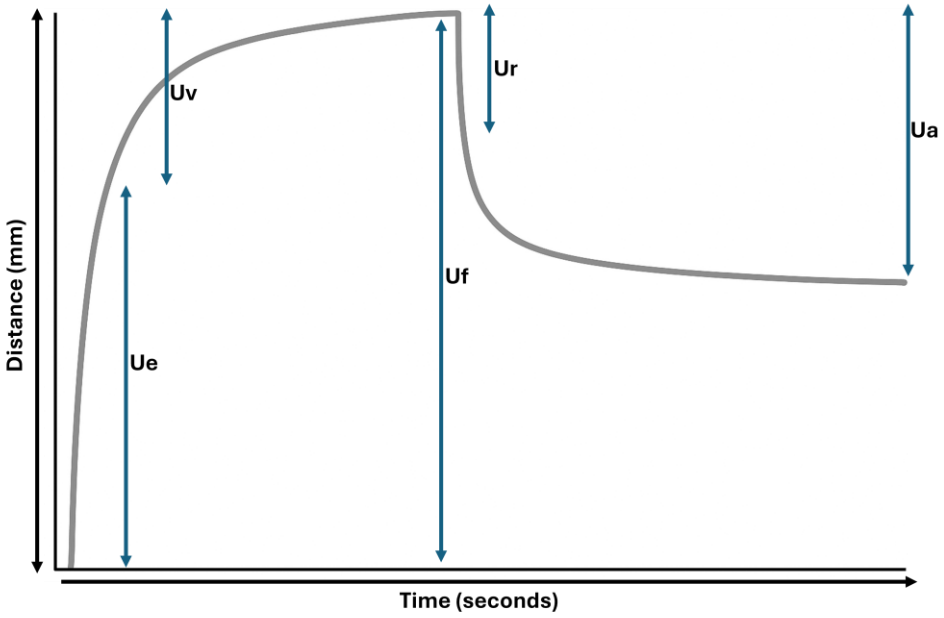

Figure S4. Visual representation of the parameters measured by the Cutometer instrument during facial skin elasticity assessments. Adapted from [58].

By tracking changes in relative parameters like R2, R5, and R7, this study can assess the effects of treatment on skin firmness and elasticity over time. In detail, R0 (Uf) measures the total passive deformation of the skin induced by suction, reflecting the amplitude at the end of the suction phase compared to the baseline measurement without suction (Figure S4). This parameter indicates the firmness or pliability of the skin. Then, R2 (Ua/Uf) represents gross elasticity, calculated as the ratio of the skin's resistance to deformation to its ability to return to its original state. Furthermore, R5 (Ur/Ue) assesses net elasticity, with higher values indicating greater skin elasticity. Finally, R7 (Ur/Uf) serves as another marker of elasticity, reflecting the skin's immediate recovery after deformation within the first 0.1 seconds [58,78].

1.7. Corneometer CM 825: Rationale and Applied Method

Skin hydration levels were measured using the Corneometer CM 825. The device operates by measuring the dielectric constant of the skin, which varies according to its water content [79]. This non-invasive method uses capacitance measurement, where changes in electrical capacitance are detected as the water content in the stratum corneum increases or decreases. Given its high sensitivity to even slight fluctuations in moisture, the Corneometer CM 825 provides precise and objective data on skin hydration dynamics [80]. Of note, the Corneometer CM 825 uses the principle of electrical capacitance, which depends on the dielectric constant of the skin. Water has a high dielectric constant relative to other skin components, allowing the device to detect even minute changes in hydration levels. When the skin is more hydrated, its dielectric constant increases, resulting in higher capacitance values [81]. Conversely, dehydrated skin exhibits lower capacitance, reflecting reduced water content. This method is particularly sensitive to the outermost

layer of the epidermis, the stratum corneum, making it ideal for tracking superficial hydration changes over time [82,83].

1.8. Photographic Recording of Treatment Zones

Photographs of both side cheek areas in controlled light conditions were taken using a Dermlite device in order to assess the appearance of dark spots at baseline D–3 and during follow-up visits at D28 and D70. Digital photographs were annotated and stored in patient files.

2. Supplementary Tables

Table S1. A description of the significance levels for global aesthetic scores.

Rating		Description
1	Very much improved	Optimal cosmetic result in this subject
2	Much improved	A marked improvement in appearance from the initial condition, but not completely optimal for this subject
3	Improved	An obvious improvement in appearance from the initial condition, but a re-treatment is indicated
4	No change	The appearance is essentially the same as the original condition
5	Worse	The appearance is worse than the original condition

Table S2. Count and percentage (n[%]) of subjects for GAIS scores, by evaluator and category. Statistical analysis was performed with a binomial test of proportion versus 0.4. GAIS, global aesthetic improvement scale.

Evaluator	Timepoint	n (%)		95% CI		*p*-value (significance)
		Improved	Not improved	Lower	Upper	
By investigator	D0 after injection	49 (100.0%)	0 (0.0%)	94.07	100	< 0.001 (S)
	D14	49 (100.0%)	0 (0.0%)	94.07	100	< 0.001 (S)
	D28	48 (100.0%)	0 (0.0%)	93.95	100	< 0.001 (S)
	D70	49 (100.0%)	0 (0.0%)	94.07	100	< 0.001 (S)
By subject	D0 after injection	46 (93.9%)	3 (6.1%)	84.93	100	< 0.001 (S)
	D14	49 (100.0%)	0 (0.0%)	94.07	100	< 0.001 (S)
	D28	48 (100.0%)	0 (0.0%)	93.95	100	< 0.001 (S)
	D70	49 (100.0%)	0 (0.0%)	94.07	100	< 0.001 (S)
Combined	D0 after injection	46 (93.9%)	3 (6.1%)	84.93	100	< 0.001 (S)
	D14	49 (100.0%)	0 (0.0%)	94.07	100	< 0.001 (S)
	D28	48 (100.0%)	0 (0.0%)	93.95	100	< 0.001 (S)
	D70	49 (100.0%)	0 (0.0%)	94.07	100	< 0.001 (S)

Table S3. Descriptive statistics and evolution over time for skin texture parameters analyzed with the Antera 3D device. When compared to D–3, a significant improvement was noted for all parameters of interest derived from image processing, characterizing texture and roughness. The improvements ranged from 10 to 18% on D28 and 7 to 13% on D70, corresponding to the 'max height (mm)' and 'texture score', respectively.

Parameter	Timepoint	n	Mean	Median	SD	Minimum	Maximum	% Variation	*p*-Value (Significance) Statistical Test Used
Texture Score	D–3	49	48.50	46.00	16.97	10.00	89.00		
	D28	48	39.78	36.50	14.92	7.00	78.50		
	D70	49	41.84	40.50	16.27	8.50	82.00		
	D28 – D–3	48	–8.85	–8.50	4.83	–24.50	–0.50	–17.98	<0.001 (S) Wilcoxon
	D70 – D–3	49	–6.66	–6.00	4.44	–19.50	–0.50	–13.74	<0.001 (S) Wilcoxon
Roughness Ra (µm)	D–3	49	10.32	9.63	2.85	4.74	18.29		
	D28	48	8.90	8.33	2.27	4.33	15.58		
	D70	49	9.23	8.92	2.52	4.56	16.52		
	D28 – D–3	48	–1.45	–1.29	0.99	–5.74	–0.15	–13.77	<0.001 (S) Wilcoxon
	D70 – D–3	49	–1.09	–1.05	0.78	–3.91	–0.03	–10.59	<0.001 (S) Wilcoxon
Roughness Rq (µm)	D–3	49	13.22	12.45	3.60	6.29	23.44		
	D28	48	11.45	10.84	2.95	5.44	20.31		
	D70	49	11.90	11.31	3.25	5.79	21.25		
	D28 – D–3	48	–1.80	–1.68	1.20	–7.06	–0.21	–13.37	<0.001 (S) Wilcoxon
	D70 – D–3	49	–1.32	–1.18	0.98	–4.76	0.15	–9.98	<0.001 (S) Paired t-test
Maximum Height (mm)	D–3	49	0.10	0.10	0.03	0.06	0.17		
	D28	48	0.09	0.09	0.02	0.04	0.15		
	D70	49	0.09	0.09	0.02	0.04	0.15		
	D28 – D–3	48	–0.01	–0.01	0.01	–0.05	0.02	–9.88	<0.001 (S) Paired t-test
	D70 – D–3	49	–0.01	–0.01	0.01	–0.03	0.02	–7.48	<0.001 (S) Paired t-test

Table S4. Descriptive statistics and evolution over time for skin texture parameters analyzed with the DermaScan device. An interesting (i.e., but not statistically supported) reduction in the 'segmented area' was noted at D28, the timepoint beyond which the mean value of the parameter remained fairly stable. 'Total intensity %' was found to be significantly reduced both at D28 and D70. 'Thickness' was found to be significantly higher at D28, but beyond that timepoint, the mean values returned to the initial state.

Parameter	Timepoint	n	Mean	Median	SD	Minimum	Maximum	% Variation	*p*-Value (Significance) Statistical Test Used
Segmented Area (mm^2)	D–3	49	6.35	6.49	2.03	2.83	11.58		
	D28	48	6.04	5.04	2.11	3.70	11.58		
	D70	49	6.08	5.45	2.20	2.76	13.27		
	D28 – D–3	48	–0.28	–0.14	1.97	–4.28	4.00	–4.85	0.332 (NS) Paired t-test
	D70 – D–3	49	–0.27	–0.01	2.00	–4.45	5.48	–4.32	0.343 (NS) Paired t-test
Total Intensity (%)	D–3	49	14.90	14.19	3.20	11.12	29.44		
	D28	48	13.46	13.39	2.56	8.54	22.01		
	D70	49	13.09	12.61	2.97	8.39	24.70		
	D28 – D–3	48	–1.48	–0.75	3.29	–8.61	5.24	–9.64	0.003 (S) Paired t-test
	D70 – D–3	49	–1.81	–1.04	3.62	–15.04	5.05	–12.13	0.002 (S) Wilcoxon
Thickness	D–3	49	1.75	1.75	0.19	1.40	2.14		
	D28	48	1.89	1.90	0.21	1.50	2.53		
	D70	49	1.70	1.72	0.18	1.26	2.04		
	D28 – D–3	48	0.15	0.14	0.10	–0.03	0.53	8.58	<0.001 (S) Wilcoxon
	D70 – D–3	49	–0.04	–0.03	0.12	–0.34	0.29	–2.48	0.016 (S) Paired t-test

Table S5. "L*" parameter (lightness) values for each subject.

Subject No.	(D–3)	(D28)	(D70)	(D28)–(D–3)	(D70)–(D–3)
S001	47.61	47.86	48.02	0.26	0.41
S002	48.46	48.89	49.56	0.43	1.10
S003	55.40	55.81	55.80	0.41	0.40
S004	54.48	55.67	57.40	1.19	2.92
S005	58.39	59.07	59.29	0.68	0.90
S006	51.45	51.60	51.68	0.14	0.22
S007	39.61	39.92	40.17	0.31	0.56
S008	53.21	54.11	54.13	0.90	0.92
S009	48.88	49.00	49.40	0.12	0.52
S010	46.05	45.95	46.08	–0.10	0.02
S011	44.59	44.41	44.67	–0.17	0.09
S012	56.82	56.87	56.34	0.04	–0.48
S013	47.05	46.94	47.83	–0.12	0.78
S014	47.09	47.46	47.83	0.37	0.75
S016	56.43	56.50	56.33	0.07	–0.10
S015	50.21	48.36	48.60	–1.85	–1.61
S040	51.01	52.07	51.36	1.05	0.34
S041	47.25	47.65	47.68	0.40	0.44
S046	45.23	45.72	45.60	0.49	0.37
S048	49.26	47.91	48.88	–1.35	–0.38
S049	49.17	48.90	49.27	–0.27	0.10
S043	47.73	47.55	46.23	–0.18	–1.50
S028	57.55	N/A	56.42	N/A	–1.14
S039	47.90	47.87	47.18	–0.03	–0.71
S038	53.76	54.23	54.67	0.47	0.90
S037	50.51	50.42	50.55	–0.08	0.04
S036	45.57	44.24	45.84	–1.33	0.27
S035	47.27	47.39	47.56	0.12	0.28
S033	50.49	49.75	50.43	–0.74	–0.06
S032	52.89	51.79	51.93	–1.09	–0.96
S031	53.97	53.76	54.79	–0.21	0.81
S022	40.58	40.16	39.90	–0.41	–0.68
S023	49.93	49.89	49.96	–0.04	0.03
S024	52.53	52.71	51.26	0.18	–1.27
S025	52.45	53.02	52.64	0.57	0.19
S026	51.60	51.81	51.69	0.21	0.09
S027	52.82	53.14	53.35	0.32	0.53
S029	48.98	48.84	48.83	–0.14	–0.15
S030	48.46	48.37	47.51	–0.10	–0.95
S034	57.17	56.04	56.68	–1.13	–0.49
S021	54.77	53.55	53.93	–1.22	–0.84
S017	46.01	45.83	45.76	–0.17	–0.24
S047	49.04	49.09	49.52	0.05	0.48
S018	51.21	51.51	51.33	0.30	0.12
S019	47.45	47.03	46.90	–0.42	–0.55
S020	54.76	54.80	54.89	0.04	0.13
S042	59.87	59.69	59.50	–0.18	–0.38
S044	38.56	38.47	39.03	–0.09	0.47
S045	39.37	39.52	39.22	0.15	–0.15

Table S6. Descriptive statistics and evolution over time for skin texture parameters analyzed with the Chromameter device. After D28 and D70, the skin appeared less red (i.e., lower a* values), with a more pronounced yellow component (i.e., higher b* values). Fairness (ITA°) remained rather stable. The general reduction (i.e., significant at D70) noted in terms of the a* parameter (i.e., skin redness), coupled with the increase in b* (i.e., yellow component) corroborate very well with improved skin fairness following the injections. Drawing from the literature, fairer subjects generally had lower levels of a* (redness), while among the darker-skinned subjects, b* was lower.

Parameter	Timepoint	n	Mean	Median	SD	Minimum	Maximum	% Variation	*p*-Value (Significance) Statistical Test Used
a*	D–3	49	13.86	13.67	1.50	10.66	18.81		
	D28	48	13.63	13.35	1.46	10.93	17.68		
	D70	49	13.26	13.22	1.39	10.77	17.37		
	D28 – D–3	48	–0.21	–0.31	0.75	–1.81	1.55	–1.67	0.056 (LS) Paired t-test
	D70 – D–3	49	–0.60	–0.70	0.96	–2.62	2.57	–4.32	<0.001 (S) Paired t-test
b*	D–3	49	18.94	19.19	1.72	14.16	22.27		
	D28	48	19.50	19.70	1.84	14.88	22.81		
	D70	49	19.50	19.57	1.71	15.12	22.62		
	D28 – D–3	48	0.51	0.52	0.96	–1.38	3.72	2.95	0.001 (S) Paired t-test
	D70 – D–3	49	0.56	0.64	0.86	–1.33	2.71	2.96	<0.001 (S) Paired t-test
ITA°	D–3	49	–0.43	–0.19	14.91	–37.56	25.33		
	D28	48	–1.15	–2.64	14.60	–37.57	26.41		
	D70	49	–0.27	–1.33	14.71	–35.02	26.74		
	D28 – D–3	48	–0.20	0.11	1.74	–5.37	2.55	167.68	0.689 (NS) Wilcoxon
	D70 – D–3	49	0.16	0.35	2.19	–4.87	6.94	–37.46	0.610 (NS) Paired t-test

Table S7. Descriptive statistics and evolution over time for skin texture parameters analyzed with the Cutometer device. A general significant increase was noted over time for all parameters assessed (i.e., whether absolute or relative). The change was more important for the relative parameters (e.g., Ua/Uf, Ur/Ue, and Ur/Uf), which is suggestive of improved skin features characterizing firmness and elasticity.

Parameter	Timepoint	n	Mean	Median	SD	Minimum	Maximum	% Variation	*p*-Value (Significance) Statistical Test Used
Ue	D–3	49	0.28	0.28	0.06	0.16	0.40		
	D28	48	0.27	0.27	0.06	0.18	0.41		
	D70	49	0.31	0.30	0.06	0.17	0.51		
	D28 – D–3	48	–0.01	0.00	0.05	–0.16	0.11	–2.65	0.320 (NS) Paired t-test
	D70 – D–3	49	0.02	0.02	0.06	–0.12	0.26	8.46	0.009 (S) Wilcoxon
Uf	D–3	49	0.36	0.36	0.06	0.25	0.49		
	D28	48	0.35	0.34	0.06	0.24	0.49		
	D70	49	0.39	0.39	0.07	0.22	0.58		
	D28 – D–3	48	–0.01	0.00	0.06	–0.19	0.13	–2.98	0.231 (NS) Paired t-test
	D70 – D–3	49	0.03	0.02	0.07	–0.13	0.27	7.81	0.005 (S) Paired t-test
Ua/Uf	D–3	49	0.78	0.80	0.08	0.61	0.92		
	D28	48	0.84	0.85	0.06	0.70	0.95		
	D70	49	0.87	0.87	0.05	0.73	0.96		
	D28 – D–3	48	0.06	0.05	0.05	–0.03	0.21	7.40	<0.001 (S) Paired t-test
	D70 – D–3	49	0.09	0.09	0.07	–0.06	0.34	11.52	<0.001 (S) Paired t-test
Ur/Ue	D–3	49	0.74	0.77	0.12	0.48	0.96		
	D28	48	0.84	0.87	0.10	0.59	1.00		
	D70	49	0.90	0.91	0.08	0.65	1.07		
	D28 – D–3	48	0.10	0.10	0.07	–0.04	0.30	14.33	<0.001 (S) Paired t-test
	D70 – D–3	49	0.16	0.14	0.10	–0.01	0.45	22.31	<0.001 (S) Paired t-test
Ur/Uf	D–3	49	0.58	0.62	0.12	0.33	0.79		
	D28	48	0.67	0.69	0.09	0.44	0.79		
	D70	49	0.71	0.72	0.08	0.49	0.87		
	D28 – D–3	48	0.08	0.08	0.07	–0.03	0.25	14.20	<0.001 (S) Paired t-test
	D70 – D–3	49	0.13	0.12	0.09	–0.05	0.38	22.35	<0.001 (S) Paired t-test

Table S8. Descriptive statistics and evolution over time for skin texture parameters analyzed with the Corneometer device. A significant increase in the hydration level was observed at all timepoints when compared to D–3.

Timepoint	n	Mean	Median	SD	Minimum	Maximum	% Variation	*p*-Value (Significance) Statistical Test Used
D–3	49	56.49	57.37	12.45	29.07	83.08		
D28	48	66.24	66.35	10.72	45.88	90.57		
D70	49	73.87	73.85	9.52	54.07	92.33		
D28 – D–3	48	10.31	9.78	3.09	4.85	21.35	17.26	<0.001 (S) Wilcoxon
D70 – D–3	49	17.39	16.30	5.40	3.83	32.52	30.78	<0.001 (S) Paired t-test
